# What Can Boron Deficiency Symptoms Tell Us about Its Function and Regulation?

**DOI:** 10.3390/plants12040777

**Published:** 2023-02-09

**Authors:** Luis Bolaños, Isidro Abreu, Ildefonso Bonilla, Juan J. Camacho-Cristóbal, María Reguera

**Affiliations:** 1 Departamento de Biología, Universidad Autónoma de Madrid, c/Darwin 2, Campus de Cantoblanco, 28049 Madrid, Spain; 2 Department of Biology, University of Oxford, South Parks Road, Oxford OX1 3RB, UK; 3 Departamento de Fisiología, Anatomía y Biología Celular, Facultad de Ciencias Experimentales, Universidad Pablo de Olavide, 41013 Sevilla, Spain

**Keywords:** boron, boron deficiency, cell signaling, plant growth and development, boron ligands, phytohormones, cell wall, reactive oxygen species (ROS), calcium signaling

## Abstract

On the eve of the 100th anniversary of Dr. Warington’s discovery of boron (B) as a nutrient essential for higher plants, “*boronists*” have struggled to demonstrate a role beyond its structural function in cell walls dimerizing pectin molecules of rhamnogalacturonan II (RGII). In this regard, B deficiency has been associated with a plethora of symptoms in plants that include macroscopic symptoms like growth arrest and cell death and biochemical or molecular symptoms that include changes in cell wall pore size, apoplast acidification, or a steep ROS production that leads to an oxidative burst. Aiming to shed light on B functions in plant biology, we proposed here a unifying model integrating the current knowledge about B function(s) in plants to explain why B deficiency can cause such remarkable effects on plant growth and development, impacting crop productivity. In addition, based on recent experimental evidence that suggests the existence of different B ligands other than RGII in plant cells, namely glycolipids, and glycoproteins, we proposed an experimental pipeline to identify putative missing ligands and to determine how they would integrate into the above-mentioned model.

## 1. Introduction

Boron (B) is a chemical element with atomic number 5. It has two isotopes, ^10^B and ^11^B, with a relative abundance of 20% and 80%, respectively, giving an atomic weight of 10.81 [1]. Together with Silicon and Germanium, B is considered a metalloid because it has intermediate properties between metals and non-metals [1,2].

It is considered a light and scarce element at Cosmos (16.9 ± 2.2 atoms/10^6^ Si), bypassed during normal thermonuclear reactions, which suggests that most B is formed by spallation reactions [1, and references therein]. Although B is widely distributed throughout the lithosphere and hydrosphere, its abundance varies in marine and terrestrial environments [3,4]. In marine environments, B concentration is approximately 0.4 mM, and it is homogenously distributed in a depth-independent (non-nutrient-like) concentration profile [4]. By contrast, in terrestrial environments, B is considered a scarce element, although its distribution here is heterogeneous, ranging from areas with deficiency to zones with concentrations that are considered toxic [3]. 

Besides being an essential element for plant growth widely studied by Plant Physiologists, during the last few years, B and B-containing minerals are attracting researchers of prebiotic chemistry. Borate was apparently essential for the early synthesis of ribofuranose [5,6], nucleic acid bases, biogenic carboxylic acids, and amino acids [7]. Furthermore, it was shown that borosilicate increased the efficiency and yielding of the Miller-Urey famous experiment [8] 

Within plants, the requirement of B is characterized by occurring within a narrow range of concentrations, out of which deficiency or toxicity symptoms begin to appear. These will be highly determined by the type of crop and edaphoclimatic conditions [3,9]. For instance, B is easily lixiviated in soils under high rainfall conditions, although the presence of organic matter increases retention of boric acid (H_3_BO_3_) by esterification with *cis*-diol-containing residues [10], a property that can be on the basis of the physiological functions of B [11], as will be stated below. 

Although Agulhon confirmed in 1910 that B existed in plants and proposed that it might be a catalytic fertilizer [12], the first studies about its essentiality began in the early 1920s when Katherine Warrington demonstrated in *Vicia faba* (and other plant species) that the absence of B led to a reduced root growth [13]. In fact, it is now known that B availability in many soils dedicated to agriculture is low, making the element the most limiting mineral micronutrient for plant productivity, perhaps with the exception of iron (Fe) and zinc (Zn). Indeed, B nutritional stress in plants affects many crops worldwide, limiting both yield quantity and quality [3,14].

During almost one century since Warrington’s studies, a plethora of biochemical, physiological, or anatomical plant processes affected by B deficiency, which we listed in the following section, have been reported and reviewed in the past years by different authors, including Brown et al. [15], Goldbach & Wimmer [16], or Camacho-Cristóbal et al. [9]. Even more, its essentiality for plant growth has been questioned with the exciting hypothesis that B is certainly toxic for plants, and plants increase tolerance via phenolic sequestering [17]. However, the fact that organisms that do not synthesize phenolics, including animals, also suffer malformations under B-starvation [18,19,20,21], in many cases shared with those occurring in plants [22], still supports its role as an essential element.

In the last years, molecular biology approaches and high-throughput methodologies have focused B research on (i) the identification of genes increasing Boron Acquisition Efficiency (BAE) and Boron Use Efficiency (BUE); (ii) the description of omic changes in response to B deficiency and toxicity; or (iii) the characterization of signalling pathways in response to B deficiency, and have proven the participation of B in plant cellular roles not necessarily related to cell wall functioning (see IJMS Special Issue: Novel Aspects of Boron Biology in Plants. Boron and Plant Interaction, 2021) [23]. 

In this review, we summarized the existing knowledge about B physiology, and the demonstrated or proposed biological roles of this micronutrient in plants based on the described B deficiency symptoms. We also considered the molecules that are proposed to be ligands of boric acid/borate in plants (and other organisms) and the physiological responses of these organisms to changing B levels. Finally, we proposed and discussed a unifying model of B functions based on the reactivity of boric acid and borate to molecules containing diol groups.

## 2. One Hundred Years of Boron Research

Since Agulhon (1910) [12], a huge number of reports have explored different aspects of B plant physiological responses, which reflect the great interest aroused by this plant mineral nutrient. During the following lines, we summarized a century of knowledge in B research and honoured the authors who have greatly contributed to it. We apologize in advance for missed contributions, as there is no space to include all of them in a very few pages.

Starting with Warrington’s studies [13], the first reports on B nutrition described that major symptoms of deficiency in plants were growth arrest, root swelling [24,25,26,27,28], or the accumulation of ‘brown’ substances, concomitant to necrosis and plant death [29,30,31]. Following the improvement of histological and biochemical techniques, different evidence that growth arrest is due to the inhibition of cell elongation [32,33] or/and arrest of cell division [34,35,36] was provided, and electron microscopy revealed defects in cell wall thickness and architecture [37].

The challenge then was to link B deficiency symptoms with its primary functions. In this regard, different studies related B with alkaloid synthesis [38,39]; indoleacetic acid metabolism [40]; gibberellic acid [41]; lignin metabolism [42]; membrane integrity [43,44]; nitrate metabolism [45,46]; nucleic acid metabolism [47,48,49]; phenol metabolism [50,51]; sucrose transport [52,53]; and sugar metabolism [54,55]. However, 80 years of B research were required to depict the primary role of B in the cell wall structure. 

### 2.1. Boron and the Cell Wall: The Only Demonstrated Primary Role

The attention of ‘*boronists*’ very soon focused on the cell wall. Katherine Warrington already indicated that B is fixed by the plant [13], and later, Skok provided evidence that B’s role was related to the complexing capacity of borate ions [56]. Mazurek & Perlin described boric acid/borate complexes with diols containing compounds [57], and Loomis & Durst reported that 90% of B is associated with the cell wall fraction [58]. Years later, Matoh et al. raised the amount of B in the cell wall up to 98% [59], and Hu & Brown localized B associated with cell wall pectin and proposed that “…B plays a critical, although poorly defined, role in the cell wall structure of higher plants” [60].

That poorly understood role of B in the cell wall began to be defined when Findeklee & Goldbach showed that the elasticity of the cell wall is reduced under B-deficiency [61], which pointed to a B function in anchoring cell wall macromolecules and to the capacity of borate cross-linking two chains of rhamnogalacturonan II (RGII) through diol-ester bonds [62]. Matoh et al. found later that such RGII-dimer (dRGII) was ubiquitous in the cell wall of higher plants [63]. Finally, O’Neill et al. convincingly demonstrated that the growth of *Arabidopsis thaliana* dwarf mutants *mur1* relies on the presence of apiose-borate complexes mediating the dimerization of RGII [64,65], which is described in detail in Section 3. Although RGII is a minor part of the pectin fraction [66], more than 90% appear dimerized, which is critical for plant growth. Specifically, this dRGII-borate complex is important to determine pore size and cell wall mechanic properties influencing cell wall expansion and, therefore, plant growth [67,68].

Although the basic structure of RGII and the B-mediated dRGII-B are highly conserved in vascular plants, some variability has been described among different plant species [69]. The relative abundance of the pectin fraction is lower in monocots than in dicots, likely explaining its lower B requirement [70]. Primitive plants contain traces of dRGII-B also with the same conserved structure, which suggests that genes involved in RGII biosynthesis appeared early in the evolution of land plants and that RGII dimerization was crucial for such evolution [71].

### 2.2. The Fine-Tuning of Boron Homeostasis. Does It Support Other Primary Roles of This Micronutrient?

Being the structural role the only demonstrated primary role of B in plants, it is proposed that many of the B deficiency symptoms result from the drastic changes in the cell wall structure and properties resulting from the decrease in dRGII-borate complexes [65]. Even more, the rapid cell death after inducing B deficiency has been attributed to defects in newly forming cell walls [72]. Nevertheless, it should not preclude the possibility that other primary functions of B may exist, particularly those related to the maintenance of membrane activities or/and regulation of developmental events, perhaps influencing cell signalling and transduction pathways [15,16,73].

Although there is still a lack of convincing evidence supporting those mechanisms underlying alternative roles of B in plant cells, the discovery of different transporters involved in B uptake and inner transport may support that this micronutrient´s role goes beyond being a cell wall structural element. 

Indeed, although most B is associated with RG-II in land plants [63], it can be found in the cytosol and vacuole [74] or associated with the plasma membrane [75,76]. Independently of the concentration of B in soils, its concentration in cell walls is kept almost constant, while B cytosolic levels vary with soil concentrations. This has resulted in the interesting hypothesis that a minimal level not bound to RG-II is needed to avoid several early B-deficiency symptoms [77] and that the reaction of the non-RG-II-linked B with other ‘ligands’ might be responsible for roles of B beyond the cell wall [11].

The uptake of B by plants was considered an unregulated passive process until the discovery of the presence of a complex transport system that was acting to maintain B homeostasis [78]. Today, B passive diffusion through the plasma membrane is considered to occur only for B uptake from soil to roots [79], but it is accompanied by transport mediated by channels [80] that have also been described in growing shoot tissues [81] and in reproductive organs [82,83]. In summary, plants sense internal and external conditions of B and rapidly regulate the expression of channels of the NIP (*Nodulin26-like Intrinsic Protein*) subfamily of MIPs (MAJOR INTRINSIC PROTEIN) family and transporters of the Borate Exporter family (BOR) to control B homeostasis [84]. In *Arabidopsis*, AtNIP5;1 is responsible for facilitating the uptake of B from soil to roots [85], and the exporter BOR1, which is localized toward the stele, is key for xylem loading under low-B conditions [86], and BOR2 seems to export B from the symplast to the apoplast to ensure efficient RG-II cross-linking [87]. Meanwhile, BOR4 is induced under high-B concentrations and is involved in the exclusion of B, enhancing B toxicity tolerance [88]. 

Additionally, other borate channels have been described to be expressed in different tissues and at particular developmental stages. *AtNIP6;1* is expressed mainly in the node region of shoots and is involved in xylem-phloem translocation of B to growing leaves [81]; AtNIP7;1 seems to be required for pollen development [83]; and *AtNIP4;1* and *AtNIP4;2* expressions are related to pollen tube elongation [89]. Moreover, evidence that X Intrinsic Proteins (-XIPs), another subfamily of MIPs, can facilitate B transport to young tissues has also been reported [90]. Altogether, the complex mechanism of B uptake and translocation must guarantee continuous supply to grow cell walls, but, at the same time, it is preferentially distributed to developing meristems, which supports that B is not a merely structural element but plays a potentially key role in developmental processes.

Reinforcing the importance of B for plant development, orthologs and paralogs of Arabidopsis BORs and NIPs have been described in many plant species [91,92,93,94,95,96,97]. Sequences similarities indicate that these transporters belong to conserved gene families that show developmental stage-dependent expression patterns in different tissues to reach B requirements that ensure the proper execution of blueprints for the plant building [84].

### 2.3. Boron and Cell Membranes

Before demonstrating B function in RGII crosslinking, researchers focused their attention on cell membranes describing that B deficiency impairs membrane transport, membrane-associated enzymatic activities, or membrane composition, and interestingly, during the last years, the evidence of B roles as a linker element in the cell membrane and endomembranes has re-emerged.

Robertson & Loughman demonstrated a reduced absorption of phosphate under B deficiency [98], and Goldbach showed that phosphate and glucose uptake and efflux rates were decreased under B-limiting conditions [99]. Also, K-Cl stimulated ATPase [43], and ATP-dependent H^+^ pumping and vanadate-sensitive ATPase activities were demonstrated to be inhibited by B deficiency [100]. In all cases, these effects were quickly reverted by B addition, suggesting that the membrane properties rely on B nutrition. Furthermore, B has also been involved in redox activities and the maintenance of membrane potential [101].

A structural role of B in the membrane was proposed by several researchers to explain a large number of reported effects of B on membrane processes. For instance, the fluidity of liposomes prepared from low B treated cells of sunflower was lower than in liposomes coming from B-sufficient cells [100]; deficiency reduced both total lipid and phospholipid contents in roots and leaves of *Lycopersicon esculentum* (tomato) and *Abelmoschus esculentus* (okra) [102], and Cakmak et al. (1995) showed increments of solutes´ leakage under B deficiency [103]. Altogether, these findings indicate that B could primarily affect membrane function by playing a structural role that protects membrane integrity. This is supported by Tanada analyses that showed a major part of B localized in membranes [104]. Nevertheless, although membranes harbour glycans as good candidates for binding B, the difficulty identifying B complexes formed with membrane components does not allow us to discard that the observed effects are secondary events of the affected cell wall.

At the end of the 20th and the beginning of the 21st Century, new techniques, and new biological models for plants, animals, and prokaryotes, have allowed the development of new approaches to unravel membrane-related B functions. For instance, the use of phenylboronic acid (PBA) as a competitor for B-binding sites caused the disassembly of transvacuolar cytoplasmic strands and cell collapse [105], suggesting either a structural role of B in the cytoskeleton or, more likely, a disruption of cytoskeletal proteins anchored to membrane glycolipids or/and glycoproteins. Also, following a similar experimental procedure, it was shown that PBA induced abnormal internalization of PIN1, blocking auxin transport and generating abnormal Arabidopsis embryo early development, which supported that PBA competes with B for membrane proteins [106].

A large amount of information relating B with membrane glycans has come from the research performed on the legume-rhizobia symbioses [107,108,109]. This symbiotic interaction triggers the development of a new organ, the root nodule, that follows a unique process of organogenesis characterized by events in which exists an intense membrane synthesis. It is estimated that the membrane synthesis rate is about 30 to 50-fold higher than in other plant-growing organs [110]. Matching with the analyses reported by Tanada, the content of B in nodules is higher than in roots or shoots, likely because it is demanded by such an amount of membrane synthesis [111]. Therefore, the legume-rhizobia symbioses is a very suitable model to investigate the role of B in membrane-located processes. The bacteria proliferate inside the nodule and differentiate into N_2_-fixing bacteroids enclosed by a plant-derived peribacteroid membrane (PBM), which differentiates a glycocalyx composed of new glycolipids and glycoproteins [112,113] involved in bacteria-plant cell surface interactions important to ensure the success of the symbiosis [114]. Some of those components are either abnormally glycosylated [107] or not detected [108] in B-deficient nodules, resulting in cell cycle and the cell division-cell differentiation transition misregulation that leads to a tumour-like development [109]. Recently, an abnormal N-glycosylation during early development under B deficiency has been described in pea nodules, Arabidopsis roots, and *Dario rerio* [22]. Like in nodules, aberrant root apical meristem in Arabidopsis and a failure of zebrafish organogenesis occurred. Although the described aberrant development could be due to defects in the cell wall structure, the effects observed in the animal model support a primary role of B in membranes, likely related to the synthesis and stability of glycan moieties of glycoproteins and glycolipids. As mentioned above, *cis*-diol-containing sugar residues, harboured by the cell glycocalyx in membranes and matrices, are potential candidates to be ligands of B. Several of them have already been identified in studies that will be described later [75,76,115].

### 2.4. Boron and Developmental Events

Back in 1985, Lovatt published an interesting hypothesis stating that the evolution of the xylem resulted in the acquisition of the essentiality of B for apical meristem activity and conferred the advantage of preventing B toxicity [116], coinciding with the recent consideration of Lewis that affirmed that B is a toxic element for vascular plants [17]. Lovatt suggested that a threshold concentration of B must reach meristematic cells to promote division and subsequent expansion to ensure growth, preventing the accumulation of B in meristems to a toxic concentration. The evolution of the xylem ensured a gradient of B, lowering its concentration in the growing cells in the elongation zone and reaching a critical minimum content in the meristematic cells to elicit mitosis. The hypothesis was supported by the fact that B is toxic to most organisms at relatively very low concentrations, being vascular plants the most tolerant, and by observing that DNA synthesis, cell division, and elongation are inhibited under B deprivation and soon reversed after B supply. The review also proposed that regulation of cell division by B may be potentially common to other organisms than vascular plants.

More recent studies have shown that induced B deficiency or loss of function mutations on B translocators alters cell cycle regulatory pathways, cell differentiation, and the development of vegetative and reproductive structures [91,117,118,119,120]. Particularly, responses to deficiency described in those and other studies recently reviewed [121,122] suggest that B can induce molecular pathways that determine meristem fate and place B nutrition as a key regulator of developmental processes.

Indeed, the earliest defects following B-starvation are growth arrest and aberrant meristem formation [119,123,124]. This growth arrest can be attributed to defects in cell elongation or differentiation due to abnormal cell wall architecture [65,119,125], although evidence of cell division inhibition has also been reported. Actually, at the initial steps of B nutrition research, the works performed by Sommer and Sorokin supported that B deficiency caused root tip malformations and impairment of cell division [29]. And as mentioned above, using PBA to mimic B-deficiency, it was shown that root apical meristem (RAM) formation was disrupted in embryos as early as the first asymmetric cell division of the hypophysis appeared [106], placing B as crucial for embryo formation. Furthermore, Poza-Viejo et al. reported a reduction of cell division 4h after transferring Arabidopsis seedlings to severe B deficiency media due to inhibition of the G1-DNA replication phase transition [120]. Cell division inhibition was accompanied by a later loss of identity of the quiescent centre (QC) that could be attributed to the down-regulation of *CCS52A2* that controls QC and the maintenance of surrounding stem cells [126].

As previously stated, the interaction of legumes with soil rhizobia triggers an interesting process of plant organogenesis in which cell division, cell elongation, and cell differentiation must be finely regulated [127,128]. Particularly crucial is the activation by CCS52A of the transition from mitosis to endoreduplication to gain polyploidy required for cell elongation prior to bacteria invasion [129]. Interestingly, *CCS52A* is also downregulated during early organogenesis of B-deficient nodules, leading to failure of cell elongation and cell differentiation [117]. Both in the QC and in nodule cells, expression of *CCS52A(A2)* promotes ubiquitination and proteolysis of the anaphase-promoting complex, resulting in cell polyploidy. This is crucial to maintain QC identity and mitotic activity of surrounding stem cells of RAM and to induce nodule cell elongation, bacterial invasion/spreading, and cell differentiation, respectively.

Development of reproductive organs is often more sensitive to B deficiency than vegetative growth [123]; therefore, it is not surprising that specific mechanisms of B homeostasis are induced at a particular moment in which shoot meristems transition from vegetative to floral development [130]. Apparently, BRAHMA (BRM) protein, which is degraded in response to high B [131], maintains the juvenile phase [132]. The transition to a mature phase prior to reproductive development may be the consequence of the reduction in BRM activity in response to the B translocation increase driven by B transporters [122]. 

Altogether, it seems that this micronutrient could play central roles in molecular regulatory pathways of the embryo and post-embryo plant development.

### 2.5. Boron, Cell Signaling Mechanisms, and Gene Expression Regulation

Recent studies using microarrays or RNAseq have provided increasing evidence supporting B nutrition’s effect on the regulation of gene expression affecting metabolism, cell wall, and membrane integrity and function, stress response, or micronutrient homeostasis [118,133,134,135,136,137]. As summarized in the following lines, almost every signalling and transduction pathway is activated in response to B deficiency, which allows us to hypothesize that the micronutrient may be involved in cell signalling. Nevertheless, many experimental data support that most of the responses are linked to the effect on cell walls. Briefly, based on our and other author´s studies, Kobayashi et al. [72] proposed that the disturbed pectin network decreases the tensile strength of the cell wall leading to an increase of turgor pressure that stretches the cell membrane triggering a stress response that resembles hypersensitive responses to pathogens. Transduction of such mechanical signal involves a rapid influx of Ca^2+^, ROS production, and MAPK cascades that result in auxin/ethylene-mediated cessation of growth and cell death [125,138,139,140,141]. This is supported by the fact that blocking Ca^2+^ channels in B-deprived cells largely inhibited the expression of stress-responsive genes [139] or adding antioxidant reagents can prevent the death of B-deficient cells [138] by restoring cell elongation. Similarly, blocking ethylene biosynthesis or perception or using mutants defective in ethylene or auxin response can restore B deficiency molecular responses [125]. Furthermore, destabilization of the cell wall under Ca deficiency but not under other nutrient deficiencies such as K or Mg that are also involved in pectin cross-linking triggers a similar response [140]. On the contrary, preincubating B-deficient cells with a supplement of extra Ca increases cell wall strengthening, attenuating the expression of B-deficiency-responsive genes [140]. Similarly, the addition of Ca partially restored the impaired development of B-deficient legume nodules [111] and the expression of 75% of genes affected by stress [118].

In roots, Ca influx, ROS production, and cell death occur preferentially in the elongation zone [72]. In line with this, maize mutants affected in the synthesis of B transporters, which are involved in the transition to the reproductive phase, develop defective inflorescences with reduced RG-II dimerization, which is largely restored by adding B into the media [91]. Therefore, failure of meristem formation and functioning can also be explained by the mechanosensitive response to B deficiency. But is that all?

Based on the comparison among different gene expression profiles, the mechanosensitive hypothesis proposes a pathogen-like response under B deficiency. However, B-deficiency disturbs cell wall structure, and it is expected that the upregulation of genes is involved in cell wall functioning. Nevertheless, while genes related to the cell wall structure, included in different transcriptomic analyses, are upregulated after pathogen attack [142], they appear downregulated under B deficiency [72,134], suggesting that B might be required to induce the expression of cell wall synthesis and assembly-related genes [74]. Using the legume nodule model, the first visible symptoms of B deficiency appear early after root inoculation with rhizobia [143], and although pathogenesis-related proteins were synthesized [144], oxidative damage was not detected even 3 weeks post-inoculation [145]. At this developmental stage, cell death is not observed, but it appears an abnormal cell division resembling tumour behaviour [109], indicating an early failure of development and suggesting that B deficiency is not necessarily associated with cell death. Furthermore, low-B results in abnormal embryonic development in animals [19], also leading to a tumour-like amorphous structure when B deprivation occurs at the early cleavage stage [22]. Interestingly, boric acid can inhibit cell proliferation in different cancer cell lines [146,147]. Such implications of B nutrition in animal physiology/development, together with the organogenesis failure in plants and animals, claim for alternative or/and additional sensing/response mechanisms to low B conditions.

The existence of a putative B sensor molecule in plants able to detect external B concentration remains still unknown. Certainly, the loss of cell wall integrity that can alter turgor pressure could be the cellular signal triggering the B stress response. In agreement with such a hypothesis, the literature offers other possible sensing mechanisms associated with soluble, not necessarily external, B. Regarding B deprivation sensing mechanisms, a computational model for B distribution in roots localized the highest concentration of soluble B around the QC, which might be likely used to keep RAM activity [148]. Also, the fact that the 5′-untranslated region (UTR) of NIP5;1 responds to the increase in cytosolic B promoting mRNA degradation [149] led to the assumption of a sensor mechanism acting in the cytosol resulting in the development of biosensors of cytosolic B [150]. Interestingly, this 5′-UTR response to B seems to function also in animal cells [149]. Additionally, there are different cell wall receptors that act in response to stresses regulating cell wall dynamics [151]. Also, the arabinogalactan-proteins (AGP) have been proposed as sensors of soluble periplasmic B because, as we described later, they contain sugar residues susceptible to interaction with B [152]. Afterwards, the signal would be conveyed to the nucleus, and several cell signalling transduction pathways can be implicated. In agreement with this hypothesis, Dumont et al. demonstrated that the inhibition of root cell elongation induced by the fucose analogue 2-fluoro 2-L-fucose (a chemical inhibitor of RG-II biosynthesis) was partially restored by boric acid supplementation without rescuing RG-II synthesis nor dimerization [153]. These observations suggest that B itself, rather than the RG-II dimer, is an essential component of the cell wall integrity-sensing mechanism that controls cell elongation, perhaps due to its ability to bind to the *cis*-diol motifs of signalling molecule(s).

The potential role of phytohormones in the regulation of B stress responses has been widely studied. By applying pharmacological approaches combined with reverse genetics using mutant lines affected in hormone synthesis or hormonal perception/transduction pathways, it was described that B deprivation alters plant development affecting synthesis, transport, or/and reception of auxins [106,154], ethylene [144,155], cytokinins [119,120,156], brassinosteroids [157], jasmonic acid [158], and the cross-talk among hormones [159]. Thus, phytohormone regulatory pathways are considered crucial in regulating cell signalling in B nutrition, although other cell signalling mechanisms common in plants and animals must also be important.

As previously mentioned, blocking sites of B binding with PBA led to cytoskeleton disruption [105], and levels of actin and tubulin increased in response to short-term B starvation due to the altered cytoskeleton polymerization [160]. Therefore, B might be involved in signalling through a cascade of signals via the cell wall-plasma membrane-cytoskeleton continuum [16], through endocytosis of signalling elicitors. Supporting this hypothesis, the abundance of homogalacturonan and RGII rapidly increased in cell walls shortly after B-deprivation in *Zea mays*, and their endocytosis was also inhibited [161]. In animals, the maintenance of the membrane-cytoskeleton continuum and, hence, endocytosis-mediated signalling also support the essentiality of B in the development of these organisms.

Also, the fact that the increase of cytosolic Ca^2+^ (cytCa^2+^) is a rapid response to B deficiency could explain why B may be involved in signalling through transduction pathways activated by Ca^2+^ [152], which could also be extended to animals. Many abiotic and biotic stresses induce an increase of cytCa^2+^ following the activation of cyclic nucleotide-gated Ca^2+^ channels (CNGCs) [162]. A plasma membrane-localized CNGC was found to be upregulated in Arabidopsis in response to B deficiency [163]. Therefore, it has been proposed that membrane sensors of B-deficiency could induce activation of CNGCs resulting in Ca^2+^ increments that could activate Ca-related proteins, such as calmodulin (CaM). Ca-CaM regulates then different transcription factors and B-responsive genes [152].

Right after discovering that B is part of a signalling molecule in bacteria (AI-2 *quorum-sensing* autoinducer) that interacts with the sensor protein LuxP [164], another tentative working hypothesis to explain the possible function of B is that it is a cellular signal itself or that it is implicated in a soluble B-complex that interacts with different transcription factors. In line with this, Kasajima et al. described that WRKY6 is a transcription factor involved in response to B deficiency in Arabidopsis [165], which also plays an important role in embryogenesis [166]. Besides, B could interact with -hydroxyl groups (OH) of amino acid residues (as serine or threonine) of different transcription factors, as in the bacterial LuxP. However, there is no evidence to date of this type of binding.

## 3. Boron Complexing Molecules

Boric acid and borate form cyclic diesters with *cis*-diols containing molecules, being the most stable those on a furanoid ring structure [57]. Prior demonstration of RGII cross-linking, Loomis & Durst postulated the existence of borate-apiose complexes in the plant cell wall, or borate-mannose/galactose links that stabilize cyanobacterial heterocyst envelope, explaining the structural roles of B and, also, the B toxicity effects as borate can form diesters with ribose of ribonucleotides and RNA [58]. After the “dRGII-B” discovery, two more findings prompted the idea that the structure or/and function of other molecules depends on their interaction with the borate. The AI-2 *quorum-sensing* autoinducer and subsequent transformation in a borate-diester furanosyl structure generate the active form complex [164], which implies that B is involved in signalling pathways; furthermore, the demonstration that borate is required for the synthesis of stable pentoses, including ribose [5], suggested that B was involved in the prebiotic synthesis of biomolecules. Later, it was described that the in vitro synthesis of ribonucleotides was enhanced by B binding to 2′ and 3′ positions [167]. Therefore, it was considered that B could be a “staple” element that stabilizes the synthesis and/or activate molecules. Thus, finding molecules whose activity/function depends on borate cross-linking is considered key to demonstrating the primary roles of B (for example, furanoses, adenylates, inositides, polysaccharides, and glycans) [11]. Along this section, we shortly reviewed the main research focused on unravelling B complexing molecules in plants (summarized in Table 1) and their relationship with B deficiency in order to link particular B-complexes with functions in plant growth and development.

### 3.1. Rhamnogalacturonan II

Rhamnogalacturonan II is, perhaps, structurally, the most complex polysaccharide identified on Earth. It has a main chain similar to homogalacturonan and four branches (named A to D) composed of 13 different residues and more than 20 different linkages. RGII requires a complex process of synthesis that involves more than 50 genes, of which only a few have been identified (for review, see [66,173]). In parallel with the characterization of RGII structure, B was identified to mediate the dimerization of two RGII molecules (dRGII-B) in different plants [62,63,174,175] and was shown its implication in the in vitro dimer formation treating, under certain conditions, monomeric RGII (mRGII) with boric acid [176]. Later, it was determined that dimerization is due to borate esters at O-2 and O-3 of the apiosyl residue of side chain A in each RG-II [177,178]. 

Once demonstrated the in vivo dRGII-borate complex formation it remained the question of whether cross-linking occurs during the synthesis of RGII in the endomembrane system, after secretion to the periplasmic space, or in muro once it is incorporated into the cell wall. Supported by in vitro boric acid mediated dimerization of mRGII obtained by acid hydrolysis of cell walls [176,177], the hypothesis of in muro dimerization was first accepted and maintained for about 15 years. Studies on cultured cells confirmed a correlation between B levels and RGII dimerization, since the addition of B to B-deprived cells increased the amount of dRGII-B, but not the amount of mRGII retained in walls of B-deficient cells [179,180]. These results suggest that dimerization occurs in newly synthesized RGII that could occur during intracellular synthesis or during secretion. Chormova et al. observed that B-deficiency acclimated cells resupplied with B form dRGII-B, but when treatment to inhibit de novo synthesis of polysaccharides preceded B resupply, dRGII was not detected despite maintaining mRGII amounts [181,182]. Therefore, the authors concluded that B-bridging must occur in Golgi or during exocytosis, but not after secretion into the apoplast. Recently, in a finely performed work using [^14^C]glucose to label the newly synthesized RGII in *Rosa* and *Arabidopsis* cell cultures, the same team concluded that B-bridging of RGII “occurs predominantly within the Golgi system, prior to release into the apoplast, and continue at a much-reduced rate after secretion” [183].

### 3.2. Cell Wall and Extracellular Matrix Glycoproteins

Although demonstrated dimerization of RGII by B seems to occur mainly in the endomembrane system, is still unclear the precise mechanism controlling the in vivo dimerization. There is huge evidence that suggests that it must be accompanied by interaction, likely mediated also by B, with the cell wall and matrix glycoproteins. Extensins and other cationic histidine-rich-, proline-hydroxyproline-rich (HPRG)-glycoproteins have been proposed as natural chaperones that facilitate the formation of dRGII-B [182]. Extensins can be located on the inner face of the cell wall, interacting with B and “waiting” for the secretion of newly synthesized RGII, perhaps as B-donors, to immediately catalyse cross-linking or, alternatively, it could be located inside the Golgi cisternae or Golgi-derived vesicles to dimerize RGII before secretion. Although it is not demonstrated that wall glycoproteins interact, at least transiently, with B, immunocytochemical analysis of *Phaseolus vulgaris* B-deficient nodules revealed that cortical cells have walls with not covalently linked HPRG [169]. Since the synthesis was not apparently affected by B deficiency, it was interpreted that B has a role in the assembly of wall components prior to secretion.

Periplasmic arabinogalactan proteins (AGPs) comprise a major and highly diverse group of plant cell matrix glycoproteins, many of which have GPI-lipid membrane anchors and have been considered analogous to extracellular matrices of animal cells (reviewed in [184,185]). The glycan moiety might act as a soluble signal and as a coreceptor of morphogens regulating embryo [186,187,188] and post-embryo pattern formation [189,190,191], and many other processes of plant growth and development [184,185]. The three mannose residues and the galactose residues of AGPs with GPI-lipid anchor are potential sites of interaction with B that have led AGPs to be postulated as sensors of B deficiency [152].

The first evidence of interaction between B and matrix glycoproteins came from using the specific boric acid-chelating resin Amberlite IRA743 [192] (which can “capture” potential molecules complexed with B) following affinity chromatography of fractions derived from *Pisum sativum* symbiotic nodules and immunostaining with specific antibodies. Besides RGII, a legume-specific glycoprotein from nodule infection threads and extracellular matrices was identified as a putative borate ligand [145]. This glycoprotein, first termed root nodule extensin (RNE), was structurally characterized as a heteropolymer that alternated glycol motifs of extensin and AGP. Later, it was renamed arabinogalactan protein-extensin (AGPE) [193]. This protein is implicated in the apical growth of infection threads which seems to be regulated by the transition from fluid to a solid state of AGPE through peroxide-driven tyrosine cross-linking at the tip growing point [194]. In B-deficient nodules, infection threads appeared swollen with inhibited apical growth and aborted prematurely [195], suggesting that the interaction of AGPE with borate is crucial. These results share similarities with the previously known requirement of B for the growth of pollen tubes, one of the most characteristic events of cell apical growth in angiosperms [196,197]. Very interestingly, AGPs secreted to the stigma, and the style are crucial for pollen tube guidance [198].

The legume AGPE structure suggests that it is covalently associated with cell wall components reinforcing the infection thread’s structure [193]. Indeed, epitope tag modifications altered its behaviour in the extracellular matrix, preventing crosslinking in the cell walls of transformed tobacco cells [199]. After the demonstration that AGPE is a potential ligand of B, co-immunoprecipitation assays allowed identifying of a stable association between AGPE and RGII in B-fed legume nodules that was not detected in B-deficient nodules [115]. Therefore, although there is still a lack of evidence of the nature and specific sites of interaction, B promotes the formation of an AGPE-RGII (likely an AGPE-B-RGII) complex that reinforces infection thread wall during growth, as might occur in pollen tubes.

### 3.3. Ligands in Cell Membranes: Glycolipids and Glycoproteins

An important amount of B was detected associated with membrane fractions [105]. Plant cell membranes have glycolipids and glycoproteins that contain different residues such as galactose, mannose, inositol, and hydroxyl-aminoacids that contain sites of potential interaction with borate. In 1977, Pollard et al. [43] hypothesized that the simplest explanation for impaired membrane functions under B deficiency is that B interacts directly with polyhydroxy components of membrane glycoproteins and glycolipids. However, the first evidence came up much later. 

By studying legume nodule development using immunohistochemistry, it was circumstantially detected that αRGII labelled the peribacteroid membranes (PBM) in B-sufficient but not in B-deficient nodules [108]. Following fractionation, three glycoproteins sharing antigenicity with RGII were identified and appeared in both PBM and cell membranes of differentiating cells and disappeared once cell differentiation was completed. The fact that antibody labelling was concentrated at the interface membrane-apoplast or PBM-peribacteroid space and that the vesicle merging that is targeted to symbiosomes fails in B-starved nodules led to postulate that B stabilizes RGII-glycoproteins in Golgi-derived vesicles and facilitates vesicle fusion during symbiosome development and nodule organogenesis [109]. Nevertheless, it was not demonstrated that those glycoproteins were B interactors.

One year later, Dr Goldbach’s group, using phenyl boronate affinity chromatography followed by 2-D electrophoresis and MALDI-TOF, isolated and identified membrane proteins able to interact in vitro with borate in root microsomal fractions of *Arabidopsis* and *Zea mays* [75]. Several of the identified proteins are especially interesting as they are related to processes affected by B deficiency. For example, the binding capacity of B to various H^+^-ATPases can be related to the early reported affected H^+^ transport through membranes [100] since B may stabilize the enzymes in the membrane; several are involved in plant defence responses, which match well with the model of defence-like response to B-deficiency described by Kobayashi et al. [72]. Particularly relevant was the identification of endoplasmic reticulum (ER) B-binding proteins, including different luminal-binding proteins such as the chaperone BiP which facilitates the assembly of protein complexes within the ER and also acts in the ER quality control mechanism that recognizes and sends to degradation abnormally folded proteins [200]. Disruption of ER homeostasis led to ER stress and to the accumulation of misfolded proteins in its lumen. Furthermore, the accumulation of N-glycosylated proteins is a general feature of the development of legume nodules, roots, and also of *Danio rerio* embryos [22]. Using Amberlite IRA-743 in affinity chromatography experiments, several linked-glycan-linked glycoproteins were isolated from legume nodules developed with optimal B nutrition but not from B-starved nodules [201], suggesting that the interaction with B is important to ensure a proper glycosylation/folding. Besides, B-sufficient nodules also yielded BiP isoforms but not from B-deficient nodules. The use of an anti-BiP antibody revealed that these proteins were synthesized and even accumulated in B-deficient legume nodules and *Arabidopsis* roots, which indicated that B-deficiency can lead to ER stress.

Concerning glycolipids, glycosylinositol phosphoryl ceramides (GIPCs) ubiquitous in plants, animals, and bacteria were circumstantially isolated and identified as B-binding glycolipids from vegetative cell membranes of the cyanobacterium Anabaena cultivated in media with B, but not in the absence of this micronutrient [202]. Also, Voxeur and Fry discovered that disruption of borate ester linkages enhanced the extractability of glycosylinositol phosphoryl ceramides (GIPCs) from Rosa cells and showed that GIPCs are able to interact with RG-II, possibly forming a GIPC-B-RGII complex that can favour the dimerization of RG-II [76]. Interestingly, GIPCs are major components of lipid rafts in eukaryotic cells, and it is proposed that the alteration of lipid rafts can be responsible for the effects of B deficiency. Lipid rafts are functionally membrane microdomains that can bind GPI-anchored, transmembrane protein or linked-glycan-linked proteins that are likely transported from the endoplasmic reticulum and assembled in the Golgi apparatus [203]. They play important roles in vesicle biosynthetic and endocytic traffic, and GPI, N-glycans, or G-proteins associated with lipid rafts can activate different signal transduction pathways, also in animal development, which can be the basis to explain the failure of plant and animal organogenesis under B deficiency.

Overall, interactions mediated by B involving RGII-extensins-AGP-GPI might be essential to maintain structurally and functionally the cell-wall-membrane-cytoskeleton continuum. Moreover, the binding of B to GIPCs and glycoprotein associated with lipid rafts apparently stabilized the function of the endomembrane system, vesicular traffic, and signal transduction pathways associated with the membranes, which may be common for plants and animals.

### 3.4. Soluble Potential Ligands

Soluble borate complexes with sorbitol, mannitol, fructose, sucrose, and N-acetyl-serine have been identified in phloem sap as mechanisms of phloem B mobility [170,171]. Fructoborates have a positive impact on human health when used as a dietary supplement of B [204]. They are considered natural sources of B to satisfy B requirements of animals and humans, but there is no evidence of their regulatory roles.

Many regulatory and/or signalling molecules are ribonucleotides with the potential capacity of forming B complexes through diesters with 2′ and 3′ OH of its ribosyl residue. Ralston and Hunt used capillary electrophoresis to identify and quantify B binding to adenosine-containing molecules and ranked it from S-adenosylmethionine (SAM) with the highest affinity for B decreasing in NAD, adenosine phosphates (ATP, ADP, AMP), and cAMP with the lowest affinity [172]. A plausible explanation of the undoubtedly beneficial effects of dietary B for human and farm animal health partially holds on the formation of adenylate-borate complexes [205], which can be common to plants and has been inherited by “plant *boronists*” as a working hypothesis. However, to date, there is no experimental evidence of in vivo borate-complexing with nucleotides. Moreover, B was hypothetically important for the stable prebiotic synthesis of ribonucleotides [167], but once synthesized, the phosphate at the 5′-position destabilizes borate bound to ribose [206]. This can explain why SAM has a higher affinity for B binding and, at the same time, does not support the in vivo complexation of ribonucleotide phosphates. SAM decreased in the liver of rats fed with poor B diets [207], and it is the precursor for bacterial AI-2 synthesis [164]. Therefore, it is a more plausible ligand of B with biological importance. Nonetheless, the potential in vivo SAM-B complexes remain still to be demonstrated. 

Other potential B ligands are phosphoinositides (PI) and soluble inositol phosphates (IP), which contain two hydroxyl groups in *cis* configuration at positions 2 and 3 [11,15,205]. Based on the observed increase in cytosolic Ca^2+^ concentration in response to B deficiency [139,152], a potential complexation of B to PIs or IPs could prevent IP3-mediated calcium release and the subsequent signalling cascade in response to B. However, to date, B-inositol complexes have not been isolated. Moreover, it is likely that the proximity of several phosphate groups destabilized B bounding, as occurs in ribonucleotides.

An important group of regulatory molecules at the post-transcriptional level are miRNAs, which are also potential ligands of B. Several reports describe that miRNAs are important regulators for the adaptation of plants to B-deficiency [208,209] and toxicity [210]. However, to our knowledge, the formation of in vivo B-miRNAs has not yet been explored. On the contrary, it has been shown that borate favours the destabilization of polymeric RNA, and therefore, it is postulated that its participation in the prebiotic synthesis should be restricted to ribonucleotides and not extended to polynucleotides [211].

Altogether, there is a lack of evidence for the essential roles of soluble-borate complexes during growth and development. On the contrary, some studies reconsider the idea of Loomis & Durst, stating that the interaction of B with ribose of ribonucleotides may explain some effects of B toxicity [58] and support that B can disrupt metabolism and development by binding to ATP, NADH or RNA [9,212,213]. Nevertheless, the ‘toxical’ binding of B to ribonucleotides could be used to achieve a beneficial effect following B resupply after a B-deficient period. Indeed, it has been demonstrated that boric acid complexed to NAD^+^ inhibits ADP-ribosyl cyclase that in eukaryotes converts NAD^+^ to cyclic-ADP-ribose (cADPR), a second messenger that triggers the release of Ca^2+^ from storage compartments [214]. This ‘toxic’ action of B inhibiting Ca^2+^ release by complexing NAD inhibits the proliferation of prostate cell cancer [215]. Hypothetically, the resupply of B to B-deficient plants could inhibit Ca^2+^ release by the same or similar mechanism, complexing cNMP precursors and inhibiting cytosolic Ca^2+^ increase through cyclic nucleotide-gated Ca^2+^ channels. This could therefore stop Ca^2+^-induced accumulation of ROS and prevent cell death that is activated in response to B deficiency, being a mechanism to return to a normal physiological status.

## 4. A unifying Model for B Function(s) in Plants and Concluding Remarks

**Figure 1:**
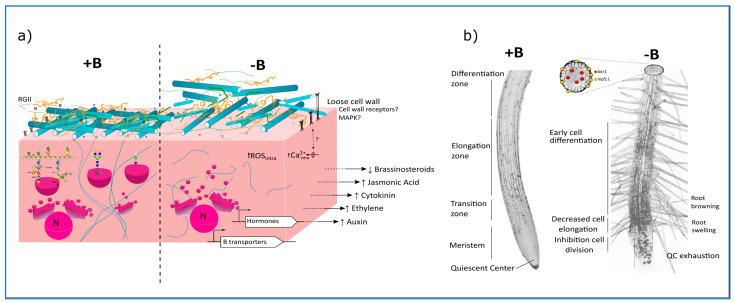
B deficiency symptoms and signalling pathways controlling B deficiency response in roots. (**a**) At the cellular level, boron sufficiency (+B) ensures the dimerization of rhamnogalacturonan II (RGII) and the bridging of other *cis*-diol-containing molecules such as glycoproteins and glycolipids, so the cytoskeleton/plasma membrane/cell wall (C/PM/CW) continuum is maintained, and root development progress normally. In B deficiency (−B), cells cannot sustain RGII dimerization nor crosslinking of other *cis*-diol-containing molecules, resulting in the accumulation of vesicles likely containing monomeric RGII and glycoproteins in the cell. In parallel, the lack of dimeric RGII would lead to the loosening of the cell wall, which perceived by specific cell wall receptors, will trigger primary a Ca2+ influx and ROS burst, and then the accumulation of the stress phytohormones ethylene and jasmonic acid, root morphogens auxins and cytokinins, and the inhibition of brassinosteroid signalling. (**b**) At the root level, the above-mentioned signalling pathways will arrest growth by targeting numerous processes in root development: exhaustion of the quiescent centre, arrest of cell division, inhibition of cell elongation, and early cell differentiation.

Boron deficiency in plants has been widely explored for 100 years at cellular, physiological, and molecular levels. Considering the main purpose of this review of proposing a unifying model for B deficiency responses and for B primary roles, we have focused on the early events triggered by B deficiency which are temporally and spatially relevant at the cell/tissue scale trying to connect potential primary B functions with potential ligands. Based on it, we summarized our proposal in Figure 1.

To date, the only undoubtedly confirmed function of B in plants is the dimerization of RGII [65], which determines cell wall porosity and strength [67,68]. How to connect this primary function of B with the signalling pathways involved in the B deficiency response? The mechanical stimuli proposed by Kobayashi et al. [72] might not be sufficient to explain the diversity of symptoms of B-deficient plant growth and development and could not be the unique sensing mechanism. The description of cell wall receptors for particular stresses [151] opens the possibility that plants could perceive specifically dRGII-B status and trigger specific responses to low borate-mediated dimerization. Among the families of cell wall receptors, wall-associated kinases (WAKs) are the best-characterized pectin receptors [216]. The induction of WAK2-dependant activation of invertase is detected after in vitro addition of RGII to protoplast, but no RGII (nor RGI) binding was detected [217]. Other cell wall receptor family interacting with pectin comprises proline-rich extensin-like receptor kinases (PERKs). They are kinases located at the plasma membrane which contain a proline-rich, extracellular domain similar to that of extensins likely embedded in the cell wall, that likely cross-link to wall extensins [218], and that are not covalently bound to B-deficient walls [169]. Many AGPs with GPI-anchors bind cell wall pectins [219], and because the GPI anchor may be cleaved by phospholipases and the AGP moiety liberated from the plasma membrane [220] could also act as cell wall sensors. Since it was shown that legume-AGPE binds B and B promotes the formation of AGPE-RGII complex [115], it should be considered a candidate to sense B-deficiency.

These cell wall receptors would transmit B deficiency through the membrane and the cytoskeleton continuum to elicit several signalling pathways, which would explain the pleiotropic effects of B deficiency on plant growth and development. Briefly, several of the B-deficiency symptoms summarized in this review could be explained by the combined activation of several phytohormone regulatory pathways, which would include, at least, jasmonic acid-dependent wound/Cell Wall Damage (CWD) response [158], ethylene, auxin, and auxin/ethylene cross-talking [106,141,154], cytokinin (CK) and auxin/CK cross-talking [120,156,221], or brassinosteroids (BR) [157].

Although not mediated by phytohormones, transduction of B-deficiency sensing through ubiquitous pathways involving Ca^2+^ and Ca^2+^-dependent activation of calmodulins would satisfactorily explain symptoms of B-starvation in plants and in organisms without RGII or devoid of cell walls [152], whenever a sensing mechanism of B not located in cell wall exists. A diversity of membrane glycolipids and/or glycoproteins common to plants, animals, and even bacteria, are already identified as potential ligands of B are good candidates [77,78,202], and their interactions with B can maintain membrane integrity and membrane transport and signalling mechanisms. The first evidence of B essentiality in animal development was dysplasia during the early cleavage period of zebrafish embryonic development, associated with membrane blebbing and cytoplasm extrusion on the animal pole [222]. Images of low B embryos resemble those when the release of extracellular vesicles (EVs) occurs and suggest uncontrolled extracellular dumping. EVs, and particularly exosomes, are naturally formed by cells, contain signalling or regulatory molecules, and participate in cell-to-cell communication [223].

EVs formation is associated with membrane lipids microdomains, or lipid rafts [224,225]. Therefore, observed membrane blebbing could be attributed to abnormal lipid raft function. Supporting this hypothesis, several glycolipids and glycoproteins with potential B-binding capacity are associated with lipid rafts [203]. Lipid rafts do not occur only in the plasma membrane but also are organized in intracellular membranes. They are essential for maintaining almost all cellular functions, including, besides EVs formation, the spatial organization of the plasma membrane, sensing and transduction, lipid, and protein trafficking from ER to Golgi apparatus, vesicle trafficking from Golgi to different target sites, and the formation of endocytic vesicles and endosome movements [224]. 

Cellular and biochemical approaches provided evidence that B is required for correct endomembrane trafficking. Short-term B deficiency led to rapid accumulation of both actin and tubulin in Arabidopsis [161]. This coincides with ultrastructural changes described in B-deficient cells as early as 1976, which included fast intracellular accumulation of swollen vesicles [226]. Abnormal accumulation of N-linked glycoproteins, which is a general feature of B deficiency in plants and in animals [22], seems to be also related to a failure of protein and glycan secretion through the endomembrane trafficking that could be related to glycolipids/glycoproteins interacting with B. 

All this evidence points to a failure of the endomembrane mechanics in response to B deficiency, which is common to plants and animals. As mentioned, N-linked high-mannose-rich glycoproteins (HMRG) accumulate during the development of Arabidopsis, legume nodules, and zebrafish embryos [22]. The HMR-glycan is added in the ER, and subsequent N-glycan processing involves trimming and substitution reactions to a more complex glycan in the ER and finally in Golgi [227]. Modifications of these N-linked HMR-glycans are crucial for correct protein folding and serve as tags for quality control [228]. Failures in N-glycan trimming lead to an accumulation of HMRG proteins in the ER, and the subsequent activation of UPR (Unfolded Protein Response) and ERAD (ER-Associated Degradation) responses, growth arrest, and root swelling [229]. All of them are symptoms observed under B-deficiency, which suggests a failure of N-glycosylation, which is supported by the literature.

Indeed, B deficiency results in the abnormal glycosylation of particular proteins crucial for cell surface interactions regulating developmental events, as occurs with PsNLEC-1, a lectin involved in symbiosome development in pea nodules [230]. This protein is accumulated as a pre-N-linked glycosylated form in cytosolic vesicles and vacuole [107], being B-deficient nodules phenotypically similar to those developed in pea mutants with altered glycosylation [231]. Other glycoproteins components of the symbiosome and plasma membrane are not detected in B-deficient nodules [108]. Indirect evidence that the phenomenon of abnormal N-glycosylation is also common to animal cells is the fact that HMRGP also accumulates in zebrafish larvae when B-deficiency was induced just prior to 60h post-egg fertilization. At that time, an intense *de novo* synthesis of glycans targeted to regions of pectoral fins, jaws, and sensorial organs preceded its development [232].

Following fractionation of legume nodules and affinity chromatography with Amberlite IRA-743, several N-linked glycoproteins were isolated as potential B-ligands that were accumulated in B-deficient nodules [201]. Among them, it was found the already described Ps-NLEC-1 and, interestingly, several forms of BiP that were also isolated by boronate affinity chromatography from Arabidopsis and *Zea mays* microsomal fractions [75], which may be indicative of an activation of UPR due to underglycosylation leading to ER stress [233]. 

After one century of studies, more recent evidence claims to draw a scenario trying to find a model for common ubiquitous responses of living forms to B deficiency. To date, the most accepted primary function of B in plants is maintaining cell wall structure by the convincingly demonstrated cross-linking of RGII [64]. Sensing the perturbed pectin network, either both mechanistically or through pectin interacting cell wall receptors triggers a response to B deficiency that is conveyed to the nucleus, affecting several regulatory pathways that would explain all the described symptoms on growth and development [72]. But sensing at the cell wall site does not explain why B deficiency also has similar effects on animal development.

Trying to solve this puzzle with the aid of the whole research revisited for this review, an alternative model that can be complementary to the cell wall sensing mechanism is based on the potential interaction of B with membrane glycolipid and glycoproteins. The accumulation of HMRG in B-deficient plants and animals is surely reflecting underglycosylation due to the instability of ER membrane and ER stress, as a common symptom of B deficiency. The first immediate consequence is that vesicle trafficking is altered. Secondly, membrane or extracellular glycans involved in cell signalling, which are crucial for plant or animal organogenesis, are not accurately targeted or, if targeted, are abnormally glycosylated. Then, cell surface sensors are not functional, the formation of plasma membrane lipid rafts fails, the (wall)-plasma membrane-cytoskeleton continuum breaks, exosome and endocytic vesicle formation is uncontrolled, and finally, induction signalling from neighbouring cells ‘short-circuits’ leading to tumour-like early development or to defective organogenesis. As a feedback effect trying to restore membrane glycans and the continuum cell surface-cytoskeleton, enhanced synthesis of glycans and cytoskeletal proteins that keep accumulating in an ER stress environment occurs.

Going back to the primary roles of B in plants, the fact that a marginal amount of mRGII is found in B-deficient cells and that the addition of borate does not promote dimerization of previously synthesized mRGII [180] is consistent with this model. Indeed, dimerization exclusively occurs in de novo synthesized pectin or during the synthesis or prior to secretion, and it is facilitated by membrane glycoproteins and glycolipids [76,168]. Therefore, although RGII-crosslinking is undoubtedly a primary role of B in plants, it might be considered that it is not the primary function since correct dRGII-B formation seems to hold on the N-glycosylation machinery, which is apparently common in all eukaryotes. Supporting this, the Arabidopsis *mur1-1* mutant, in which RG-II crosslinking was demonstrated essential for plant growth [64], shows defects in N-glycosylation accumulating HMGP despite a lack of fucose-complex N-glycans [234]. In this mutant, although boric acid or fucose partially restored growth, it did not reach wild-type rosette development [64], indicating the importance of N-glycosylation for dRGII-dimer formation and secretion.

To conclude this review, we proposed to further investigate the proposed role of B on N-glycosylation and secretion mechanisms and their relationship with the synthesis, complexing, secretion, and correct deposition of RGII. Arabidopsis reporter lines affected in different cell components’ secretion have already been developed [235,236] and combining these tools with immunocytochemical approaches may be useful to validate and contextualize the failure of secretion of glycoproteins and/or dRGII-B under B deficiency. Also, it would be interesting to analyse the changes in N-glycosylation in the *mur1-1* mutant line and other mutants affected by N-glycosylation in relation to B nutrition, which might help to identify and to confirm the existence of an in vivo linking of B to glycan residues that might be crucial for a proper N-glycosylation. In parallel, similar approaches on animal or animal cell lines would provide further knowledge of the common primary functions of B in N-glycosylation mechanisms in plants and animals. 

## Figures and Tables

**Table 1:** Boron binding molecules in plants.

Ligand	Location	Function	Source
Rhamnogalaturonan II (RGII)	Cell wall matrix (Golgi vesicles, Golgi)	Cell wall structure stability	[64,65]
Histidine/proline-hydroxyproline-rich glycoproteins (HPRGs)	Cell wall matrix	Regulation of cell extension	[168,169]
Arabinogalactan proteins (AGPs)	Cell wall matrix	Cell wall dynamics, cell signaling	[115,145]
Membrane glycoproteins and glycolipids	Endoplasmic reticulum, Golgi, Mitochondria, Cell membrane	Membrane dynamics, cell signaling, transport, metabolism	[75,76]
Sugars, Polyols	Phloem sap	Boron mobility	[170,171]
Ribonucleotides	Cytosol	Signal transduction	[172]
Phosphoinositide (PIP), inositol phosphates (IPs)	Cell membranes, cytosol	Signal transduction	Putative [11]

## Data Availability

Not applicable.

## References

[1] Power P.P., Woods W.G. (1997). The Chemistry of Boron and Its Speciation in Plants. Plant Soil.

[2] Broadley M., Brown P., Cakmak I., Rengel Z., Zhao F., Marschner P. (2012). Function of Nutrients: Micronutrients. Marschner’s Mineral Nutrition of Higher Plants.

[3] Shorrocks V.M. (1997). The Occurrence and Correction of Boron Deficiency. Plant Soil.

[4] Carrano C.J., Schellenberg S., Amin S.A., Green D.H., Küpper F.C. (2009). Boron and Marine Life: A New Look at an Enigmatic Bioelement. Mar. Biotechnol..

[5] Ricardo A., Carrigan M.A., Olcott A.N., Benner S.A. (2004). Borate Minerals Stabilize Ribose. Science.

[6] Scorei R. (2012). Is Boron a Prebiotic Element? A Mini-Review of the Essentiality of Boron for the Appearance of Life on Earth. Orig. Life Evol. Biosph..

[7] Saladino R., Barontini M., Cossetti C., Di Mauro E., Crestini C. (2011). The Effects of Borate Minerals on the Synthesis of Nucleic Acid Bases, Amino Acids and Biogenic Carboxylic Acids from Formamide. Orig. Life Evol. Biosph..

[8] Criado-Reyes J., Bizzarri B.M., García-Ruiz J.M., Saladino R., Di Mauro E. (2021). The Role of Borosilicate Glass in Miller–Urey Experiment. Sci. Rep..

[9] Camacho-Cristóbal J.J., Rexach J., González-Fontes A. (2008). Boron in Plants: Deficiency and Toxicity. J. Integr. Plant Biol..

[10] Goldberg S. (1997). Reactions of Boron with Soils. Plant Soil.

[11] Bolaños L., Lukaszewski K., Bonilla I., Blevins D. (2004). Why Boron?. Plant Physiol. Biochem..

[12] Agulhon H. (1910). The Presence and Utility of Boron in Plants. Ann. Inst. Pasteur.

[13] Warington K. (1923). The Effect of Boric Acid and Borax on the Broad Bean and Certain Other Plants. Ann. Bot..

[14] Nable R.O., Bañuelos G.S., Paull J.G. (1997). Boron Toxicity. Plant Soil.

[15] Brown P.H., Bellaloui N., Wimmer M.A., Bassil E.S., Ruiz J., Hu H., Pfeffer H., Dannel F., Römheld V. (2002). Boron in Plant Biology. Plant Biol..

[16] Goldbach H.E., Wimmer M.A. (2007). Boron in Plants and Animals: Is There a Role beyond Cell-Wall Structure?. J. Plant Nutr. Soil Sci..

[17] Lewis D.H. (2019). Boron: The Essential Element for Vascular Plants That Never Was. New Phytol..

[18] Bonilla I., Garcia-González M., Mateo P. (1990). Boron Requirement in Cyanobacteria Its Possible Role in the Early Evolution of Photosynthetic Organisms. Plant Physiol..

[19] Rowe R.I., Eckhert C.D. (1999). Boron Is Required for Zebrafish Embryogenesis. J. Exp. Biol..

[20] Bolaños L., Redondo-Nieto M., Bonilla I., Wall L.G. (2002). Boron Requirement in the Discaria Trinervis (Rhamnaceae) and Frankia Symbiotic Relationship. Its Essentiality for Frankia BCU110501 Growth and Nitrogen Fixation. Physiol. Plant..

[21] Fort D.J., Rogers R.L., McLaughlin D.W., Sellers C.M., Schlekat C.L. (2002). Impact of Boron Deficiency on Xenopus Laevis: A Summary of Biological Effects and Potential Biochemical Roles. Biol. Trace Elem. Res..

[22] Reguera M., Abreu I., Sentís C., Bonilla I., Bolaños L. (2019). Altered Plant Organogenesis under Boron Deficiency Is Associated with Changes in High-Mannose N-Glycan Profile That Also Occur in Animals. J. Plant Physiol..

[23] González-Fontes A., Fujiwara T. IJMS. Special Issue: Novel Aspects of Boron Biology in Plants. Boron and Plant Interaction. https://www.mdpi.com/journal/ijms/special_issues/plant_boron.

[24] Sommer A.L., Lipman C.B. (1926). Evidence on the Indispensable Nature of Zinc and Boron for Higher Green Plants. Physiology.

[25] Johnston E.S., Dore W.H. (1929). The Influence of Boron on the Chemical Composition and Growth of the Tomato Plant. Plant Physiol..

[26] McHargue J.S., Calfee R.K. (1933). Further Evidence That Boron Is Essential for the Growth of Lettuce. Plant Physiol..

[27] Neales T.F. (1959). The Boron Requirement of Flax Roots Grown in Sterile Culture. J. Exp. Bot..

[28] Dear J., Aronoff S. (1965). Relative Kinetics of Chlorogenic and Caffeic Acids During the Onset of Boron Deficiency in Sunflower. Plant Physiol..

[29] Sommer A.L., Sorokin H. (1928). Effects of the Absence of Boron and of Some Other Essential Elements on the Cell and Tissue Structure of the Root Tips of *Pisum sativum*. Plant Physiol..

[30] Spurr A.R. (1952). Fluorescence in Ultraviolet Light in the Study of Boron Deficiency in Celery. Science.

[31] Whittington W.J. (1959). The Role of Boron in Plant Growth: The Effect on Growth of the Radicle. J. Exp. Bot..

[32] Odhnoff C. (1957). Boron Deficiency, and Growth. Physiol. Plant..

[33] Albert L.S., Wilson C.M. (1961). Effect of Boron on Elongation of Tomato Root Tips. Plant Physiol..

[34] Cohen M.S., Albert L.S. (1974). Autoradiographic Examination of Meristems of Intact Boron-deficient Squash Roots Treated with Tritiated Thymidine. Physiology.

[35] Cohen M.S., Lepper R. (1977). Effect of Boron on Cell Elongation and Division in Squash Roots. Plant Physiol..

[36] Moore H.M., Hirsch A.M. (1983). Effects of Boron Deficiency on Mitosis and Incorporation of Tritiated Thymidine into Nuclei of Sunflower Root Tips. Amer. J. Bot..

[37] Spurr A.R. (1957). Boron in Morphogenesis of Plant Cell Walls. Science.

[38] Steinberg R.A. (1955). Effect of Boron Deficiency on Nicotine Formation in Tobacco. Plant Physiol..

[39] Tso T.C., McMurtrey J.E., Jeffrey R.N. (1962). Mineral Deficiency & Organic Constituents in Tobacco Plants. III. Plant Growth & Alkaloid Contents Related to Gradual Development of Calcium or Boron Deficiency Symptoms. Plant Physiol..

[40] Hirsch A.M., Pengelly W.L., Torrey J.G., Torreyt J.G. (1982). Endogenous IAA Levels in Boron-Deficient and Control Root Tips of Sunflower. Bot. Gaz..

[41] Skok J. (1968). Relationship of Boron to Gibberellic Acid-Induced Proliferation in Debudded Tobacco Plants. Plant Physiol..

[42] Lewis D.H. (1980). Boron, Lignification and the Origin of Vascular Plants. A Unified Hypothesis. New Phytol..

[43] Pollard A.S., Parr A.J., Loughman B.C. (1977). Boron in Relation to Membrane Function in Higher Plants. J. Exp. Bot..

[44] Cakmak I., Römheld V. (1997). Boron Deficiency-Induced Impairments of Cellular Functions in Plants. Plant Soil.

[45] Bonilla I., Cadahía C., Carpena O., Hernando V. (1980). Effects of Boron on Nitrogen Metabolism and Sugar Levels of Sugar Beet. Plant Soil.

[46] Camacho-Cristóbal J.J., González-Fontes A. (1999). Boron Deficiency Causes a Drastic Decrease in Nitrate Content and Nitrate Reductase Activity and Increases the Content of Carbohydrates in Leaves from Tobacco Plants. Planta.

[47] Chapman K.S.R., Jackson J.F. (1974). Increased RNA Labelling in Boron-Deficient Root-Tip Segments. Phytochemistry.

[48] Wainwright I.M., Palmer R.L., Dugger W.M. (1980). Pyrimidine Pathway in Boron-Deficient Cotton Fiber. Plant Physiol..

[49] Lovatt C.J., Albert L.S., Tremblay G.C. (1981). Synthesis, Salvage, and Catabolism of Uridine Nucleotides in Boron-Deficient Squash Roots. Plant Physiol..

[50] Watanabe R., Chorney W., Skok J., Wender S.H. (1964). Effect of Boron Deficiency on Polyphenol Production in the Sunflower. Phytochemistry.

[51] Ruiz J.M., Bretones G., Baghour M., Ragala L., Belakbir A., Romero L. (1998). Relationship between Boron and Phenolic Metabolism in Tobacco Leaves. Phytochemistry.

[52] Gauch H.G., Dugger W.M. (1953). The Role of Boron in the Translocation of Sucrose. Plant Physiol..

[53] Sisler E.C., Dugger W.M., Gauch H.G. (1956). The Role of Boron in the Translocation of Organic Compounds in Plants. Plant Physiol..

[54] Halsey D., Ching F.T., Dugger W.M., Humphreys T.E. (1960). Influence of Boron on Enzymatic Reactions Associated with Biosynthesis of Sucrose. Plant Physiol..

[55] Yih R.Y., Clark H.E. (1965). Carbohydrate and Protein Content of Boron-Deficient Tomato Root Tips in Relation to Anatomy and Growth. Plant Physiol..

[56] Skok J. (1957). The Substitution of Complexing Substances for Boron in Plant Growth. Plant Physiol..

[57] Mazurek X.I., Perlin A.S. (1963). Borate Complexing by Five-Membered-Ring Vic-Diols Vapor Pressure Equilibrium and N.M.R. Spectral Observations. Can. J. Chem..

[58] Loomis W.D., Durst R.W. (1992). Chemistry and Biology of Boron. Biofactors.

[59] Matoh T., Ishigaki K.I., Mizutani M., Matsunaga W., Takabe K. (1992). Boron Nutrition of Cultured Tobacco BY-2 Cells: I. Requirement for and Intracellular Localization of Boron and Selection of Cells That Tolerate Low Levels of Boron. Plant Cell Physiol..

[60] Hu H., Brown P.H. (1994). Localization of Boron in Cell Walls of Squash and Tobacco and Its Association with Pectin (Evidence for a Structural Role of Boron in the Cell Wall). Plant Physiol.

[61] Findeklee P., Goldbach H.E. (1996). Rapid Effects of Boron Deficiency on Cell Wall Elasticity Modulus in *Cucurbita pepo* Roots. Bot. Acta.

[62] Kobayashi M., Matoh T., Azuma J.I. (1996). Two Chains of Rhamnogalacturonan II Are Cross-Linked by Borate-Diol Ester Bonds in Higher Plant Cell Walls. Plant Physiol..

[63] Matoh T., Kawaguchi S., Kobayashi M. (1996). Ubiquity of a Borate-Rhamnogalacturonan II Complex in the Cell Walls of Higher Plants. Plant Cell Physiol..

[64] O’Neill M.A., Eberhard S., Albersheim P., Darvill A.G. (2001). Requirement of Borate Cross-Linking of Cell Wall Rhamnogalacturonan II for Arabidopsis Growth. Science.

[65] O’Neill M.A., Ishii T., Albersheim P., Darvill A.G. (2004). Rhamnogalacturonan II: Structure and Function of a Borate Cross-Linked Cell Wall Pectic Polysaccharide. Annu. Rev. Plant Biol..

[66] Atmodjo M.A., Hao Z., Mohnen D. (2013). Evolving Views of Pectin Biosynthesis. Annu. Rev. Plant Biol..

[67] Fleischer A., O’neill M.A., Ehwald R. (1999). The Pore Size of Non-Graminaceous Plant Cell Walls Is Rapidly Decreased by Borate Ester Cross-Linking of the Pectic Polysaccharide Rhamnogalacturonan II. Plant Physiol..

[68] Ryden P., Sugimoto-Shirasu K., Smith A.C., Findlay K., Reiter W.D., McCann M.C. (2003). Tensile Properties of Arabidopsis Cell Walls Depend on Both a Xyloglucan Cross-Linked Microfibrillar Network and Rhamnogalacturonan II-Borate Complexes. Plant Physiol..

[69] Pabst M., Fischl R.M., Brecker L., Morelle W., Fauland A., Köfeler H., Altmann F., Léonard R. (2013). Rhamnogalacturonan II Structure Shows Variation in the Side Chains Monosaccharide Composition and Methylation Status within and across Different Plant Species. Plant J..

[70] Hu H., Brown P.H., Labavitch J.M. (1996). Species Variability in Boron Requirement Is Correlated with Cell Wall Pectin. J. Exp. Bot..

[71] Matsunaga T., Ishii T., Matsumoto S., Higuchi M., Darvill A., Albersheim P., O’Neill M.A. (2004). Occurrence of the Primary Cell Wall Polysaccharide Rhamnogalacturonan II in Pteridophytes, Lycophytes, and Bryophytes. Implications for the Evolution of Vascular Plants. Plant Physiol..

[72] Kobayashi M., Miyamoto M., Matoh T., Kitajima S., Hanano S., Sumerta I.N., Narise T., Suzuki H., Sakurai N., Shibata D. (2018). Mechanism Underlying Rapid Responses to Boron Deprivation in Arabidopsis Roots. Soil Sci. Plant Nutr..

[73] González-Fontes A., Rexach J., Navarro-Gochicoa M.T., Herrera-Rodríguez M.B., Beato V.M., Maldonado J.M., Camacho-Cristóbal J.J. (2008). Is Boron Involved Solely in Structural Roles in Vascular Plants?. Plant Signal. Behav..

[74] Dannel F., Pfeffer H., Romheld V. (1998). Compartmentation of Boron in Roots and Leaves of Sunflower as Affected by Boron Supply. J. Plant Physiol..

[75] Wimmer M.A., Lochnit G., Bassil E., Muhling K.H., Goldbach H.E. (2009). Membrane-Associated, Boron-Interacting Proteins Isolated by Boronate Affinity Chromatography. Plant Cell Physiol..

[76] Voxeur A., Fry S.C. (2014). Glycosylinositol Phosphorylceramides from Rosa Cell Cultures Are Boron-Bridged in the Plasma Membrane and Form Complexes with Rhamnogalacturonan II. Plant J..

[77] Goldbach H.E., Wimmer M.A., Findeklee P. (2000). Discussion Paper: Boron—How Can the Critical Level Be Defined?. J. Plant Nutr. Soil Sci..

[78] Miwa K., Fujiwara T. (2010). Boron Transport in Plants: Co-Ordinated Regulation of Transporters. Ann. Bot..

[79] Hu H., Brown P.H. (1997). Absorption of Boron by Plant Roots. Plant Soil.

[80] Dordas C., Chrispeels M.J., Brown P.H. (2000). Permeability and Channel-Mediated Transport of Boric Acid across Membrane Vesicles Isolated from Squash Roots. Plant Physiol..

[81] Tanaka M., Wallace I.S., Takano J., Roberts D.M., Fujiwara T. (2008). NIP6;1 Is a Boric Acid Channel for Preferential Transport of Boron to Growing Shoot Tissues in Arabidopsis. Plant Cell.

[82] Huang L., Bell R.W., Dell B. (2008). Evidence of Phloem Boron Transport in Response to Interrupted Boron Supply in White Lupin (*Lupinus albus* L. Cv. Kiev Mutant) at the Reproductive Stage. J. Exp. Bot..

[83] Routray P., Li T., Yamasaki A., Yoshinari A., Takano J., Choi W.G., Sams C.E., Roberts D.M. (2018). Nodulin Intrinsic Protein 7;1 Is a Tapetal Boric Acid Channel Involved in Pollen Cell Wall Formation. Plant Physiol..

[84] Onuh A.F., Miwa K. (2021). Regulation, Diversity and Evolution of Boron Transporters in Plants. Plant Cell Physiol..

[85] Takano J., Wada M., Ludewig U., Schaaf G., Von Wirén N., Fujiwara T. (2006). The Arabidopsis Major Intrinsic Protein NIP5;1 Is Essential for Efficient Boron Uptake and Plant Development under Boron Limitation. Plant Cell.

[86] Takano J., Noguchi K., Yasumori M., Kobayashi M., Gajdos Z., Miwa K., Hayashi H., Yoneyama T., Fujiwara T. (2002). Arabidopsis Boron Transporter for Xylem Loading. Nature.

[87] Miwa K., Wakuta S., Takada S., Ide K., Takano J., Naito S., Omori H., Matsunaga T., Fujiwara T. (2013). Roles of BOR2, a Boron Exporter, in Cross Linking of Rhamnogalacturonan II and Root Elongation under Boron Limitation in Arabidopsis. Plant Physiol..

[88] Miwa K., Takano J., Omori H., Seki M., Shinozaki K., Fujiwara T. (2007). Plants Tolerant of High Boron Levels. Science.

[89] Di Giorgio J.A.P., Bienert G.P., Ayub N.D., Yaneff A., Barberini M.L., Mecchia M.A., Amodeo G., Soto G.C., Muschietti J.P. (2016). Pollen-Specific Aquaporins NIP4;1 and NIP4;2 Are Required for Pollen Development and Pollination in *Arabidopsis thaliana*. Plant Cell.

[90] Bienert M.D., Muries B., Crappe D., Chaumont F., Bienert G.P. (2019). Overexpression of X Intrinsic Protein 1;1 in *Nicotiana tabacum* and Arabidopsis Reduces Boron Allocation to Shoot Sink Tissues. Plant Direct.

[91] Durbak A.R., Phillips K.A., Pike S., O’Neill M.A., Mares J., Gallavotti A., Malcomber S.T., Gassmann W., McSteen P. (2014). Transport of Boron by the Tassel-Less1 Aquaporin Is Critical for Vegetative and Reproductive Development in Maize. Plant Cell.

[92] Hanaoka H., Uraguchi S., Takano J., Tanaka M., Fujiwara T. (2014). OsNIP3;1, a Rice Boric Acid Channel, Regulates Boron Distribution and Is Essential for Growth under Boron-Deficient Conditions. Plant J..

[93] Pommerrenig B., Diehn T.A., Bienert G.P. (2015). Metalloido-Porins: Essentiality of Nodulin 26-like Intrinsic Proteins in Metalloid Transport. Plant Sci..

[94] Wakuta S., Mineta K., Amano T., Toyoda A., Fujiwara T., Naito S., Takano J. (2015). Evolutionary Divergence of Plant Borate Exporters and Critical Amino Acid Residues for the Polar Localization and Boron-Dependent Vacuolar Sorting of AtBOR1. Plant Cell Physiol..

[95] Granado-Rodríguez S., Bolaños L., Reguera M. (2020). MtNIP5;1, a Novel *Medicago truncatula* Boron Diffusion Facilitator Induced under Deficiency. BMC Plant Biol..

[96] Ozyigit I.I., Filiz E., Saracoglu I.A., Karadeniz S. (2020). Exploration of Two Major Boron Transport Genes BOR1 and NIP5;1 in the Genomes of Different Plants. Biotechnol. Biotechnol. Equip..

[97] Wang S., Liu L., Zou D., Huang Y., Zhao Z., Ding G., Cai H., Wang C., Shi L., Xu F. (2021). Vascular Tissue-Specific Expression of BnaC4.BOR1;1c, an Efflux Boron Transporter Gene, Is Regulated in Response to Boron Availability for Efficient Boron Acquisition in *Brassica napus*. Plant Soil.

[98] Robertson G.A., Loughman B.C. (1974). Reversible Effects of Boron on the Absorption and Incorporation of Phosphate in *Vicia faba* L.. New Phytol..

[99] Goldbach H. (1985). Influence of Boron Nutrition on Net Uptake and Efflux of (32)P and (14)C-Glucose in *Helianthus annuus* Roots and Cell Cultures of *Daucus carota*. J. Plant Physiol..

[100] Ferrol N., Belver A., Roldán M., Rodriguez-Rosales M.P., Donaire J.P. (1993). Effects of Boron on Proton Transport and Membrane Properties of Sunflower (*Helianthus annuus* L.) Cell Microsomes. Plant Physiol..

[101] Lawrence K., Bhalla P., Misra P.C. (1995). Changes in NAD(P)H-Dependent Redox Activities in Plasmalemma-Enriched Vesicles Isolated from Boron- and Zinc-Deficient Chick Pea Roots. J. Plant Physiol..

[102] Desiraju S., Sah R., Rathore V.S. (1993). Influence of Boron Deficiency on Growth, Protein and Lipid Contents in Tomato and Okra Seedlings. Acta Physiol. Plant..

[103] Cakmak I., Kurz H., Marschner H. (1995). Short-Term Effects of Boron, Germanium and High Light Intensity on Membrane Permeability in Boron Deficient Leaves of Sunflower. Physiol. Plant..

[104] Tanada T. (1983). Localization of Boron in Membranes. J. Plant Nutr..

[105] Bassil E., Hu H., Brown P.H. (2004). Use of Phenylboronic Acids to Investigate Boron Function in Plants. Possible Role of Boron in Transvacuolar Cytoplasmic Strands and Cell-to-Wall Adhesion. Plant Physiol..

[106] Matthes M., Torres-Ruiz R.A. (2016). Boronic Acid Treatment Phenocopies Monopteros by Affecting PIN1 Membrane Stability and Polar Auxin Transport in *Arabidopsis ahaliana* Embryos. Development.

[107] Bolaños L., Cebrián A., Redondo-Nieto M., Rivilla R., Bonilla I. (2001). Lectin-like Glycoprotein PsNLEC-1 Is Not Correctly Glycosylated and Targeted in Boron-Deficient Pea Nodules. Mol. Plant-Microbe Interact..

[108] Redondo-Nieto M., Pulido L., Reguera M., Bonilla I., Bolaños L. (2007). Developmentally Regulated Membrane Glycoproteins Sharing Antigenicity with Rhamnogalacturonan II Are Not Detected in Nodulated Boron Deficient *Pisum sativum*. Plant Cell Environ..

[109] Redondo-Nieto M., Reguera M., Bonilla I., Bolaños L. (2008). Boron Dependent Membrane Glycoproteins in Symbiosome Development and Nodule Organogenesis: A Model for a Common Role of Boron in Organogenesis. Plant Signal. Behav..

[110] Robertson J.G., Lyttleton P. (1984). Division of Peribacteroid Membranes in Root Nodules of White Clover. J. Cell Sci..

[111] Redondo-Nieto M., Wilmot A.R., El-Hamdaoui A., Bonilla I., Bolaños L. (2003). Relationship between Boron and Calcium in the N_2_-Fixing Legume-Rhizobia Symbiosis. Plant Cell Environ..

[112] Perotto S., Vandenbosch K.A., Butcher G.W., Brewin N.J. (1991). Molecular Composition and Development of the Plant Glycocalyx Associated with the Peribacteroid Membrane of Pea Root Nodules. Development.

[113] Perotto S., Donovan N., Drobak B.K., Brewin N.J. (1995). Differential Expression of a Glycosyl Inositol Phospholipid Antigen on the Peribacteroid Membrane during Pea Nodule Development. Mol. Plant-Microbe Interact..

[114] Bolaños L., Redondo-Nieto M., Rivilla R., Brewin N.J., Bonilla I. (2004). Cell Surface Interactions of Rhizobium Bacteroids and Other Bacterial Strains with Symbiosomal and Peribacteroid Membrane Components from Pea Nodules. Mol. Plant-Microbe Interact..

[115] Reguera M., Abreu I., Brewin N.J., Bonilla I., Bolaños L. (2010). Borate Promotes the Formation of a Complex between Legume AGP-Extensin and Rhamnogalacturonan II and Enhances Production of Rhizobium Capsular Polysaccharide during Infection Thread Development in *Pisum sativum* Symbiotic Root Nodules. Plant Cell Environ..

[116] Lovatt C.J. (1985). Evolution of Xylem Resulted in a Requirment for Boron in the Apical Meristems of Vascular Plants. New Phytol..

[117] Reguera M., Espí A., Bolaños L., Bonilla I., Redondo-Nieto M. (2009). Endoreduplication before Cell Differentiation Fails in Boron-Deficient Legume Nodules. Is Boron Involved in Signalling during Cell Cycle Regulation?. New Phytol..

[118] Redondo-Nieto M., Maunoury N., Mergaert P., Kondorosi E., Bonilla I., Bolaños L. (2012). Boron and Calcium Induce Major Changes in Gene Expression during Legume Nodule Organogenesis. Does Boron Have a Role in Signalling?. New Phytol..

[119] Abreu I., Poza L., Bonilla I., Bolaños L. (2014). Boron Deficiency Results in Early Repression of a Cytokinin Receptor Gene and Abnormal Cell Differentiation in the Apical Root Meristem of *Arabidopsis thaliana*. Plant Physiol. Biochem..

[120] Poza-Viejo L., Abreu I., González-García M.P., Allauca P., Bonilla I., Bolaños L., Reguera M. (2018). Boron Deficiency Inhibits Root Growth by Controlling Meristem Activity under Cytokinin Regulation. Plant Sci..

[121] Matthes M.S., Robil J.M., McSteen P. (2020). From Element to Development: The Power of the Essential Micronutrient Boron to Shape Morphological Processes in Plants. J. Exp. Bot..

[122] Alexandros Petropoulos S., Jiang C., Araújo W.L., Leal Pereira G., Antonio Siqueira J., Batista-Silva W., Barcellos Cardoso F., Nunes-Nesi A. (2021). Boron: More Than an Essential Element for Land Plants?. Front. Plant Sci..

[123] Dell B., Huang L. (1997). Physiological Response of Plants to Low Boron. Plant Soil.

[124] Li K., Kamiya T., Fujiwara T. (2015). Differential Roles of PIN1 and PIN2 in Root Meristem Maintenance Under Low-B Conditions in *Arabidopsis thaliana*. Plant Cell Physiol..

[125] Camacho-Cristóbal J.J., Martín-Rejano E.M., Herrera-Rodríguez M.B., Navarro-Gochicoa M.T., Rexach J., González-Fontes A. (2015). Boron Deficiency Inhibits Root Cell Elongation via an Ethylene/Auxin/ROS-Dependent Pathway in Arabidopsis Seedlings. J. Exp. Bot..

[126] Vanstraelen M., Baloban M., Da Ines O., Cultrone A., Lammens T., Boudolf V.R., Brown S.C., De Veylder L., Mergaert P., Kondorosi E. (2009). APC/CCCS52A Complexes Control Meristem Maintenance in the Arabidopsis Root. Proc. Natl. Acad. Sci. USA.

[127] Foucher F., Kondorosi E. (2000). Cell Cycle Regulation in the Course of Nodule Organogenesis in Medicago. Plant Mol. Biol..

[128] Kondorosi E., Redondo-Nieto M., Kondorosi A. (2005). Ubiquitin-Mediated Proteolysis. To Be in the Right Place at the Right Moment during Nodule Development. Plant Physiol..

[129] Vinardell J.M., Fedorova E., Cebolla A., Kevei Z., Horvath G., Kelemen Z., Tarayre S., Roudier F., Mergaert P., Kondorosi A. (2003). Endoreduplication Mediated by the Anaphase-Promoting Complex Activator CCS52A Is Required for Symbiotic Cell Differentiation in *Medicago truncatula* Nodules. Plant Cell.

[130] Diehn T.A., Bienert M.D., Pommerrenig B., Liu Z., Spitzer C., Bernhardt N., Fuge J., Bieber A., Richet N., Chaumont F. (2019). Boron Demanding Tissues of *Brassica napus* Express Specific Sets of Functional Nodulin26-like Intrinsic Proteins and BOR1 Transporters. Plant J..

[131] Sakamoto T., Tsujimoto-Inui Y., Sotta N., Hirakawa T., Matsunaga T.M., Fukao Y., Matsunaga S., Fujiwara T. (2018). Proteasomal Degradation of BRAHMA Promotes Boron Tolerance in Arabidopsis. Nat. Commun..

[132] Xu Y., Guo C., Zhou B., Li C., Wang H., Zheng B., Ding H., Zhu Z., Peragine A., Cui Y. (2016). Regulation of Vegetative Phase Change by SWI2/SNF2 Chromatin Remodeling ATPase BRAHMA. Plant Physiol..

[133] Kobayashi M., Mutoh T., Matoh T. (2004). Boron Nutrition of Cultured Tobacco BY-2 Cells. IV. Genes Induced under Low Boron Supply. J. Exp. Bot..

[134] Camacho-Cristóbal J.J., Rexach J., Herrera-Rodríguez M.B., Navarro-Gochicoa M.T., González-Fontes A. (2011). Boron Deficiency and Transcript Level Changes. Plant Sci..

[135] Koshiba T., Kobayashi M., Matsuoka K., Fujiwara T., Matoh T. (2013). Boron Nutrition of Cultured Tobacco BY-2 Cells. VII. Rapid Induction of Metabolic Dysfunction in Cells Deprived of Boron as Revealed by Microarray Analysis. Soil Sci. Plant Nutr..

[136] Lu Y.B., Qi Y.P., Yang L.T., Lee J., Guo P., Ye X., Jia M.Y., Li M.L., Chen L.S. (2015). Long-Term Boron-Deficiency-Responsive Genes Revealed by CDNA-AFLP Differ between Citrus Sinensis Roots and Leaves. Front. Plant Sci..

[137] Zhou G.F., Liu Y.Z., Sheng O., Wei Q.J., Yang C.Q., Peng S.A. (2015). Transcription Profiles of Boron-Deficiency-Responsive Genes in Citrus Rootstock Root by Suppression Subtractive Hybridization and CDNA Microarray. Front. Plant Sci..

[138] Koshiba T., Kobayashi M., Matoh T. (2009). Boron Nutrition of Tobacco BY-2 Cells. V. Oxidative Damage Is the Major Cause of Cell Death Induced by Boron Deprivation. Plant Cell Physiol..

[139] Koshiba T., Kobayashi M., Ishihara A., Matoh T. (2010). Boron Nutrition of Cultured Tobacco BY-2 Cells. VI. Calcium Is Involved in Early Responses to Boron Deprivation. Plant Cell Physiol..

[140] Oiwa Y., Kitayama K., Kobayashi M., Matoh T. (2013). Boron Deprivation Immediately Causes Cell Death in Growing Roots of *Arabidopsis thaliana* (L.) Heynh. Soil Sci. Plant Nutr..

[141] González-Fontes A., Herrera-Rodríguez M.B., Martín-Rejano E.M., Navarro-Gochicoa M.T., Rexach J., Camacho-Cristóbal J.J. (2016). Root Responses to Boron Deficiency Mediated by Ethylene. Front. Plant Sci..

[142] Bellincampi D., Cervone F., Lionetti V. (2014). Plant Cell Wall Dynamics and Wall-Related Susceptibility in Plant-Pathogen Interactions. Front. Plant Sci..

[143] Redondo-Nieto M., Rivilla R., El-Hamdaoui A., Bonilla I., Bolaños L. (2001). Boron Deficiency Affects Early Infection Events in the Pea-Rhizobium Symbiotic Interaction. Aust. J. Plant Physiol..

[144] Reguera M., Bonilla I., Bolaños L. (2010). Boron Deficiency Results in Induction of Pathogenesis-Related Proteins from the PR-10 Family during the Legume-Rhizobia Interaction. J. Plant Physiol..

[145] Reguera M., Wimmer M., Bustos P., Goldbach H.E., Bolaños L., Bonilla I. (2010). Ligands of Boron in *Pisum sativum* Nodules Are Involved in Regulation of Oxygen Concentration and Rhizobial Infection. Plant Cell Environ..

[146] Meacham S., Elwell K., Ziegler S., Carper S., Xu F., Goldbach H., Brown P., Bell R., Fujiwara T., Hunt C., Goldberg S., Shi L. (2007). Boric Acid Inhibits Cell Growth in Breast and Prostate Cancer Cell Lines. Advances in Plant and Animal Boron Nutrition.

[147] Yusuf Z.S., Uysal T.K., Simsek E., Nocentini A., Osman S.M., Supuran C.T., Özensoy Güler Ö. (2022). The Inhibitory Effect of Boric Acid on Hypoxia-Regulated Tumour-Associated Carbonic Anhydrase IX. J. Enzyme Inhib. Med. Chem..

[148] Shimotohno A., Sotta N., Sato T., De Ruvo M., Marée A.F.M., Grieneisen V.A., Fujiwara T. (2015). Mathematical Modeling and Experimental Validation of the Spatial Distribution of Boron in the Root of *Arabidopsis thaliana* Identify High Boron Accumulation in the Tip and Predict a Distinct Root Tip Uptake Function. Plant Cell Physiol..

[149] Tanaka M., Sotta N., Yamazumi Y., Yamashita Y., Miwa K., Murota K., Chiba Y., Hirai M.Y., Akiyama T., Onouchi H. (2016). The Minimum Open Reading Frame, AUG-Stop, Induces Boron-Dependent Ribosome Stalling and MRNA Degradation. Plant Cell.

[150] Fukuda M., Wakuta S., Kamiyo J., Fujiwara T., Takano J. (2018). Establishment of Genetically Encoded Biosensors for Cytosolic Boric Acid in Plant Cells. Plant J..

[151] Humphrey T.V., Bonetta D.T., Goring D.R. (2007). Sentinels at the Wall: Cell Wall Receptors and Sensors. New Phytol..

[152] González-Fontes A., Navarro-Gochicoa M.T., Camacho-Cristóbal J.J., Herrera-Rodríguez M.B., Quiles-Pando C., Rexach J. (2014). Is Ca^2+^ Involved in the Signal Transduction Pathway of Boron Deficiency? New Hypotheses for Sensing Boron Deprivation. Plant Sci..

[153] Dumont M., Lehner A., Bardor M., Burel C., Vauzeilles B., Lerouxel O., Anderson C.T., Mollet J.C., Lerouge P. (2015). Inhibition of Fucosylation of Cell Wall Components by 2-Fluoro 2-Deoxy-l-Fucose Induces Defects in Root Cell Elongation. Plant J..

[154] Li Q., Liu Y., Pan Z., Xie S., Peng S.A. (2016). Boron Deficiency Alters Root Growth and Development and Interacts with Auxin Metabolism by Influencing the Expression of Auxin Synthesis and Transport Genes. Biotechnol. Biotechnol. Equip..

[155] Tabata R., Kamiya T., Shigenobu S., Yamaguchi K., Yamada M., Hasebe M., Fujiwara T., Sawa S. (2013). Identification of an EMS-Induced Causal Mutation in a Gene Required for Boron-Mediated Root Development by Low-Coverage Genome Re-Sequencing in Arabidopsis. Plant Signal. Behav..

[156] Pommerrenig B., Faber M., Hajirezaei M., von Wirén N., Bienert G.P. (2022). Cytokinins as Boron Deficiency Signals to Sustain Shoot Development in Boron-Efficient Oilseed Rape. Physiol. Plant..

[157] Zhang C., He M., Wang S., Chu L., Wang C., Yang N., Ding G., Cai H., Shi L., Xu F. (2021). Boron Deficiency-Induced Root Growth Inhibition Is Mediated by Brassinosteroid Signalling Regulation in Arabidopsis. Plant J..

[158] Chen X., Humphreys J.L., Ru Y., He Y., Wu F., Mai J., Li M., Li Y., Shabala S., Yu M. (2022). Jasmonate Signaling and Remodeling of Cell Wall Metabolism Induced by Boron Deficiency in Pea Shoots. Environ. Exp. Bot..

[159] Eggert K., von Wirén N. (2017). Response of the Plant Hormone Network to Boron Deficiency. New Phytol..

[160] Yu Q., Wingender R., Schulz M., Baluška F., Goldbach H.E. (2001). Short-Term Boron Deprivation Induces Increased Levels of Cytoskeletal Proteins in Arabidopsis Roots. Plant Biol..

[161] Yu Q., Hlavacka A., Matoh T., Volkmann D., Menzel D., Goldbach H.E., Baluška F. (2002). Short-Term Boron Deprivation Inhibits Endocytosis of Cell Wall Pectins in Meristematic Cells of Maize and Wheat Root Apices. Plant Physiol..

[162] Yuen C.C.Y., Christopher D.A. (2013). The Group IV-A Cyclic Nucleotide-Gated Channels, CNGC19 and CNGC20, Localize to the Vacuole Membrane in *Arabidopsis thaliana*. AoB Plants.

[163] Quiles-Pando C., Rexach J., Navarro-Gochicoa M.T., Camacho-Cristóbal J.J., Herrera-Rodríguez M.B., González-Fontes A. (2013). Boron Deficiency Increases the Levels of Cytosolic Ca(2+) and Expression of Ca(2+)-Related Genes in *Arabidopsis thaliana* Roots. Plant Physiol. Biochem..

[164] Chen X., Schauder S., Potier N., Van Dorsselaer A., Pelczer I., Bassler B.L., Hughson F.M. (2002). Structural Identification of a Bacterial Quorum-Sensing Signal Containing Boron. Nature.

[165] Kasajima I., Ide Y., Yokota Hirai M., Fujiwara T. (2010). WRKY6 Is Involved in the Response to Boron Deficiency in *Arabidopsis thaliana*. Physiol. Plant..

[166] Lagacé M., Matton D.P. (2004). Characterization of a WRKY Transcription Factor Expressed in Late Torpedo-Stage Embryos of *Solanum chacoense*. Planta.

[167] Franco A., da Silva J.A.L. (2021). Boron in Prebiological Evolution. Angew Chem. Int. Ed. Engl..

[168] Chormova D., Fry S.C. (2016). Boron Bridging of Rhamnogalacturonan-II Is Promoted in Vitro by Cationic Chaperones, Including Polyhistidine and Wall Glycoproteins. New Phytol..

[169] Bonilla I., Mergold-Villaseñor C., Campos M.E., Sánchez N., Pérez H., López L., Castrejón L., Sánchez F., Cassab G.I. (1997). The Aberrant Cell Walls of Boron-Deficient Bean Root Nodules Have No Covalently Bound Hydroxyproline-/Proline-Rich Proteins. Plant Physiol..

[170] Hu H., Penn S.C., Lebrilla C.B., Brown P.H. (1997). Lsolation and Characterization of Soluble Boron Complexes in Higher Plants’ The Mechanism of Phloem Mobility of Boron. Plant Physiol..

[171] Stangoulis J., Tate M., Graham R., Bucknall M., Palmer L., Boughton B., Reid R. (2010). The Mechanism of Boron Mobility in Wheat and Canola Phloem. Plant Physiol..

[172] Ralston N.V.C., Hunt C.D. (2001). Diadenosine Phosphates and S-Adenosylmethionine: Novel Boron Binding Biomolecules Detected by Capillary Electrophoresis. Biochim. Biophys. Acta.

[173] Funakawa H., Miwa K. (2015). Synthesis of Borate Cross-Linked Rhamnogalacturonan II. Front. Plant Sci..

[174] Ishii T., Matsunaga T. (1996). Isolation and Characterization of a Boron-Rhamnogalacturonan-II Complex from Cell Walls of Sugar Beet Pulp. Carbohydr. Res..

[175] Kaneko S., Ishii T., Matsunaga T. (1997). A Boron-Rhamnogalacturonan-II Complex from Bamboo Shoot Cell Walls. Phytochemistry.

[176] O’Neill M.A., Warrenfeltz D., Kates K., Pellerin P., Doco T., Darvill A.G., Albersheim P. (1996). Rhamnogalacturonan-II, a Pectic Polysaccharide in the Walls of Growing Plant Cell, Forms a Dimer That Is Covalently Cross-Linked by a Borate Ester. In Vitro Conditions for the Formation and Hydrolysis of the Dimer. J. Biol. Chem..

[177] Ishii T., Matsunaga T., Pellerin P., O’Neill M.A., Darvill A., Albersheim P. (1999). The Plant Cell Wall Polysaccharide Rhamnogalacturonan II Self-Assembles into a Covalently Cross-Linked Dimer. J. Biol. Chem..

[178] Ishii T., Ono H. (1999). NMR Spectroscopic Analysis of the Borate Diol Esters of Methyl Apiofuranosides. Carbohydr. Res..

[179] Kobayashi M., Ohno K., Matoh T. (1997). Boron Nutrition of Cultured Tobacco BY-2 Cells. 2. Characterization of the Boron-Polysaccharide Complex. Plant Cell Physiol..

[180] Matoh T., Takasaki M., Kobayashi M., Takabe K. (2000). Boron Nutrition of Cultured Tobacco BY-2 Cells. III. Characterization of the Boron-Rhamnogalacturonan II Complex in Cells Acclimated to Low Levels of Boron. Plant Cell Physiol..

[181] Chormova D., Messenger D.J., Fry S.C. (2014). Boron Bridging of Rhamnogalacturonan-II, Monitored by Gel Electrophoresis, Occurs during Polysaccharide Synthesis and Secretion but Not Post-Secretion. Plant J..

[182] Chormova D., Messenger D.J., Fry S.C. (2014). Rhamnogalacturonan-II Cross-Linking of Plant Pectins via Boron Bridges Occurs during Polysaccharide Synthesis and/or Secretion. Plant Signal. Behav..

[183] Begum R.A., Fry S.C. (2022). Boron Bridging of Rhamnogalacturonan-II in Rosa and Arabidopsis Cell Cultures Occurs Mainly in the Endo-Membrane System and Continues at a Reduced Rate after Secretion. Ann. Bot..

[184] Seifert G.J., Roberts K. (2007). The Biology of Arabinogalactan Proteins. Annu. Rev. Plant Biol..

[185] Ellis M., Egelund J., Schultz C.J., Bacic A. (2010). Arabinogalactan-Proteins: Key Regulators at the Cell Surface?. Plant Physiol..

[186] Stacey N.J., Roberts K., Knox J.P. (1990). Patterns of Expression of the JIM4 Arabinogalactan-Protein Epitope in Cell Cultures and during Somatic Embryogenesis in *Daucus carota* L.. Planta.

[187] Chapman A., Blervacq A.S., Vasseur J., Hilbert J.L. (2000). Arabinogalactan-Proteins in Cichorium Somatic Embryogenesis: Effect of Beta-Glucosyl Yariv Reagent and Epitope Localisation during Embryo Development. Planta.

[188] Van Hengel A.J., Van Kammen A., De Vries S.C. (2002). A Relationship between Seed Development, Arabinogalactan-Proteins (AGPs) and the AGP Mediated Promotion of Somatic Embryogenesis. Physiol. Plant..

[189] Casero P.J., Casimiro I., Knox J.P. (1998). Occurrence of Cell Surface Arabinogalactan-Protein and Extensin Epitopes in Relation to Pericycle and Vascular Tissue Development in the Root Apex of Four Species. Planta.

[190] Van Hengel A.J., Roberts K. (2003). AtAGP30, an Arabinogalactan-Protein in the Cell Walls of the Primary Root, Plays a Role in Root Regeneration and Seed Germination. Plant J..

[191] Motose H., Sugiyama M., Fukuda H. (2004). A Proteoglycan Mediates Inductive Interaction during Plant Vascular Development. Nature.

[192] Asad A., Bell R.W., Dell B., Huang L. (1997). Development of a Boron Buffered Solution Culture System for Controlled Studies of Plant Boron Nutrition. Plant Soil.

[193] Brewin N.J. (2004). Plant Cell Wall Remodelling in the Rhizobium–Legume Symbiosis. Crit. Rev. Plant Sci..

[194] Wisniewski J.P., Rathbun E.A., Knox J.P., Brewin N.J. (2000). Involvement of Diamine Oxidase and Peroxidase in Insolubilization of the Extracellular Matrix: Implications for Pea Nodule Initiation by Rhizobium Leguminosarum. Mol. Plant-Microbe Interact..

[195] Bolaños L., Brewin N.J., Bonilla I. (1996). Effects of Boron on Rhizobium-Legume Cell-Surface Interactions and Nodule Development. Plant Physiol..

[196] Dickinson D.B. (1978). Influence of Borate and Pentaerythritol Concentrations on Germination and Tube Growth of Lilium Longiflorum Pollen. J. Am. Soc. Hortic. Sci..

[197] Robbertse P.J., Lock J.J., Stoffberg E., Coetzer L.A. (1990). Effect of Boron on Directionality of Pollen Tube Growth in Petunia and Agapanthus. South Afr. J. Bot..

[198] Cheung A.Y., Wang H., Wu H. (1995). ming A Floral Transmitting Tissue-Specific Glycoprotein Attracts Pollen Tubes and Stimulates Their Growth. Cell.

[199] Gucciardo S., Rathbun E.A., Shanks M., Jenkyns S., Mak L., Durrant M.C., Brewin N.J. (2005). Epitope Tagging of Legume Root Nodule Extensin Modifies Protein Structure and Crosslinking in Cell Walls of Transformed Tobacco Leaves. Mol. Plant-Microbe Interact..

[200] Ma Y., Hendershot L.M. (2004). ER Chaperone Functions during Normal and Stress Conditions. J. Chem. Neuroanat..

[201] Abreu I. (2016). New Targets in Plant Boron Deficiency Response: N-Glycosylation and Regulation of Root Development. Ph.D. Thesis.

[202] Abreu I., Orús I., Bolaños L., Bonilla I. (2014). The Interaction of Boron with Glycolipids Is Required to Increase Tolerance to Stresses in Anabaena PCC 7120. Phytochemistry.

[203] Simons K., Ikonen E. (1997). Functional Rafts in Cell Membranes. Nature.

[204] Hunter J.M., Nemzer B.V., Rangavajla N., Biţă A., Rogoveanu O.C., Neamţu J., Scorei I.R., Bejenaru L.E., Rău G., Bejenaru C. (2019). The Fructoborates: Part of a Family of Naturally Occurring Sugar–Borate Complexes—Biochemistry, Physiology, and Impact on Human Health: A Review. Biol. Trace Elem. Res..

[205] Nielsen F.H. (2014). Update on Human Health Effects of Boron. J. Trace Elem. Med. Biol..

[206] Kim D.H., Faull K.F., Norris A.J., Eckhert C.D. (2004). Borate–Nucleotide Complex Formation Depends on Charge and Phosphorylation State. J. Mass Spectrom..

[207] Nielsen F.H. (2009). Boron Deprivation Decreases Liver S-Adenosylmethionine and Spermidine and Increases Plasma Homocysteine and Cysteine in Rats. J. Trace Elem. Med. Biol..

[208] Lu Y.B., Yang L.T., Qi Y.P., Li Y., Li Z., Chen Y.B., Huang Z.R., Chen L.S. (2014). Identification of Boron-Deficiency-Responsive MicroRNAs in *Citrus sinensis* Roots by Illumina Sequencing. BMC Plant Biol..

[209] Yang C., Liu T., Bai F., Wang N., Pan Z., Yan X., Peng S.A. (2015). MiRNAome Analysis Associated with Anatomic and Transcriptomic Investigations Reveal the Polar Exhibition of Corky Split Vein in Boron Deficient *Citrus sinensis*. Mol. Genet. Genom..

[210] Huang J.H., Lin X.J., Zhang L.Y., Wang X.D., Fan G.C., Chen L.S. (2019). MicroRNA Sequencing Revealed Citrus Adaptation to Long-Term Boron Toxicity through Modulation of Root Development by MiR319 and MiR171. Int. J. Mol. Sci..

[211] Cossetti C., Crestini C., Saladino R., di Mauro E. (2010). Borate Minerals and RNA Stability. Polymers.

[212] Reid R.J., Hayes J.E., Post A., Stangoulis J.C.R., Graham R.D. (2004). A Critical Analysis of the Causes of Boron Toxicity in Plants. Plant. Cell Environ..

[213] Reid R., Xu F., Goldbach H., Brown P., Bell R., Fujiwara T., Hunt C., Goldberg S., Shi L. (2007). Update on Boron Toxicity and Tolerance in Plants. Advances in Plant and Animal Boron Nutrition.

[214] Kim D.H., Hee S.Q., Norris A.J., Faull K.F., Eckhert C.D. (2006). Boric Acid Inhibits Adenosine Diphosphate-Ribosyl Cyclase Non-Competitively. J. Chromatogr. A.

[215] Barranco W.T., Kim D.H., Stella S.L., Eckhert C.D. (2009). Boric Acid Inhibits Stored Ca^2+^ Release in DU-145 Prostate Cancer Cells. Cell Biol. Toxicol..

[216] Kohorn B.D., Kohorn S.L. (2012). The Cell Wall-Associated Kinases, WAKs, as Pectin Receptors. Front. Plant Sci..

[217] Kohorn B.D., Johansen S., Shishido A., Todorova T., Martinez R., Defeo E., Obregon P. (2009). Pectin Activation of MAP Kinase and Gene Expression Is WAK2 Dependent. Plant J..

[218] Nakhamchik A., Zhao Z., Provart N.J., Shiu S.H., Keatley S.K., Cameron R.K., Goring D.R. (2004). A Comprehensive Expression Analysis of the Arabidopsis Proline-Rich Extensin-like Receptor Kinase Gene Family Using Bioinformatic and Experimental Approaches. Plant Cell Physiol..

[219] Serpe M.D., Nothnagel E.A. (1995). Fractionation and Structural Characterization of Arabinogalactan-Proteins from the Cell Wall of Rose Cells. Plant Physiol..

[220] Borner G.H.H., Lilley K.S., Stevens T.J., Dupree P. (2003). Identification of Glycosylphosphatidylinositol-Anchored Proteins in Arabidopsis. A Proteomic and Genomic Analysis. Plant Physiol..

[221] Wang G., Römheld V., Li C., Bangerth F. (2006). Involvement of Auxin and CKs in Boron Deficiency Induced Changes in Apical Dominance of Pea Plants (*Pisum sativum* L.). J. Plant Physiol..

[222] Eckhert C.D., Rowe R.I. (1999). Embryonic Dysplasia and Adult Retinal Dystrophy in Boron-Deficient Zebrafish. J. Trace Elem. Exp. Med..

[223] Doyle L.M., Wang M.Z. (2019). Overview of Extracellular Vesicles, Their Origin, Composition, Purpose, and Methods for Exosome Isolation and Analysis. Cells.

[224] Ouweneel A.B., Thomas M.J., Sorci-Thomas M.G. (2020). The Ins and Outs of Lipid Rafts: Functions in Intracellular Cholesterol Homeostasis, Microparticles, and Cell Membranes. J. Lipid Res..

[225] Skryabin G.O., Komelkov A.V., Savelyeva E.E., Tchevkina E.M. (2020). Lipid Rafts in Exosome Biogenesis. Biochemistry.

[226] Kouchi H., Kumazawa K. (1976). Anatomical Responses of Root Tips to Boron Deficiency. Soil Sci. Plant Nutr..

[227] Kukuruzinska M.A., Lennon K. (1998). Protein N-Glycosylation: Molecular Genetics and Functional Significance. Crit. Rev. Oral Biol. Med..

[228] Helenius A., Aebi M. (2001). Intracellular Functions of N-Linked Glycans. Science.

[229] Liebminger E., Hüttner S., Vavra U., Fischl R., Schoberer J., Grass J., Blaukopf C., Seifert G.J., Altmann F., Mach L. (2009). Class I α-Mannosidases Are Required for N-Glycan Processing and Root Development in *Arabidopsis thaliana*. Plant Cell.

[230] Sherrier D.J., Borisov A.Y., Tikhonovich I.A., Brewin N.J. (1997). Immunocytological Evidence for Abnormal Symbiosome Development in Nodules of the Pea Mutant Line Sprint2Fix−(Sym31). Protoplasma.

[231] Dahiya P., Sherrier D.J., Kardailsky I.V., Borisov A.Y., Brewin N.J. (1998). Symbiotic Gene Sym31 Controls the Presence of a Lectinlike Glycoprotein in the Symbiosome Compartment of Nitrogen-Fixing Pea Nodules. Mol. Plant-Microbe Interact..

[232] Laughlin S.T., Baskin J.M., Amacher S.L., Bertozzi C.R. (2008). In Vivo Imaging of Membrane-Associated Glycans in Developing Zebrafish. Science.

[233] Nagashima Y., von Schaewen A., Koiwa H. (2018). Function of N-Glycosylation in Plants. Plant Sci..

[234] Rayon C., Cabanes-Macheteau M., Loutelier-Bourhis C., Salliot-Maire I., Lemoine J., Reiter W.D., Lerouge P., Faye L. (1999). Characterization of N-Glycans from Arabidopsis. Application to a Fucose-Deficient Mutant. Plant Physiol..

[235] Zheng H., Kunst L., Hawes C., Moore I. (2004). A GFP-Based Assay Reveals a Role for RHD3 in Transport between the Endoplasmic Reticulum and Golgi Apparatus. Plant J..

[236] Teh O.K., Moore I. (2007). An ARF-GEF Acting at the Golgi and in Selective Endocytosis in Polarized Plant Cells. Nature.

